# Acute and long-term exercise differently modulate plasma levels of oxylipins, endocannabinoids, and their analogues in young sedentary adults: A sub-study and secondary analyses from the ACTIBATE randomized controlled-trial

**DOI:** 10.1016/j.ebiom.2022.104313

**Published:** 2022-10-27

**Authors:** Lucas Jurado-Fasoli, Xinyu Di, Guillermo Sanchez-Delgado, Wei Yang, Francisco J. Osuna-Prieto, Lourdes Ortiz-Alvarez, Elke Krekels, Amy C. Harms, Thomas Hankemeier, Milena Schönke, Concepcion M. Aguilera, Jose M. Llamas-Elvira, Isabelle Kohler, Patrick C.N. Rensen, Jonatan R. Ruiz, Borja Martinez-Tellez

**Affiliations:** a PROmoting FITness and Health Through Physical Activity Research Group (PROFITH), Department of Physical Education and Sports, Faculty of Sport Sciences, Sport and Health University Research Institute (iMUDS), University of Granada, Granada, Spain; b Department of Physiology, Faculty of Medicine, University of Granada, Granada, Spain; c Department of Systems Biomedicine and Pharmacology, Leiden Academic Centre for Drug Research (LACDR), Leiden University, Leiden, the Netherlands; d Pennington Biomedical Research Center, Baton Rouge, LA 70808, USA; e Department of Analytical Chemistry, University of Granada, Granada, Spain; f Research and Development of Functional Food Centre (CIDAF), Granada, Spain; g Department of Biochemistry and Molecular Biology II, School of Pharmacy, University of Granada, Granada, Spain; h Department of Medicine, Division of Endocrinology, and Einthoven Laboratory for Experimental Vascular Medicine, Leiden University Medical Centre, Leiden, the Netherlands; i Department of Biochemistry and Molecular Biology II, “José Mataix Verdú” Institute of Nutrition and Food Technology (INYTA), Biomedical Research Centre (CIBM), University of Granada, Granada 18016, Spain; j Instituto de Investigación Biosanitaria, ibs.Granada, Granada, Spain; k CIBER Fisiopatología de la Obesidad y la Nutrición (CIBEROBN), Madrid 28029, Spain; l Servicio de Medicina Nuclear, Hospital Universitario Virgen de las Nieves, Granada, Spain; m Division of BioAnalytical Chemistry, Vrije Universiteit Amsterdam, Amsterdam Institute of Molecular and Life Sciences (AIMMS), Amsterdam, the Netherlands; n Center for Analytical Sciences Amsterdam, Amsterdam, the Netherlands; o Department of Education, Faculty of Education Sciences and SPORT Research Group (CTS-1024), CERNEP Research Center, University of Almería, Almería, Spain

**Keywords:** Aerobic, Strength, Bioactive lipids, Concurrent training, Inflammation resolution

## Abstract

**Background:**

Fatty acid-derived lipid mediators including oxylipins, endocannabinoids (eCBs), and their analogues, have emerged as key metabolites in the inflammatory and immune response to physiological stressors.

**Methods:**

This report was based on a sub-study and secondary analyses the ACTIBATE single-center unblinded randomized controlled trial (ClinicalTrials.gov ID: NCT02365129). The study was performed in the Sport and Health University Research Institute and the Virgen de las Nieves University Hospital of the University of Granada. Eligible participants were young, sedentary adults with no chronic diseases. Here, we performed both an acute endurance and resistance exercise sub-studies (n = 14 and 17 respectively), and a 24-week supervised exercise intervention, combining endurance and resistance exercise training at moderate-intensity (MOD-EX) or vigorous-intensity (VIG-EX) exercise groups, in young sedentary adults. Randomization was performed by unrestricted randomization. Plasma levels of oxylipins, eCBs, and their analogues were measured using liquid chromatography-tandem mass spectrometry.

**Findings:**

Both endurance and resistance exercise increased by +50% the plasma levels of dihomo-γ-linolenic acid and arachidonic acid (AA) omega-6 derived oxylipins, as well as eicosapentaenoic acid and docosahexaenoic acid omega-3 derived after 3 and 120 min of the bout of exercise (all η^2^ ≥ 0.219 and P ≤ 0.039). These exercise modalities also increased the levels of anandamide and eCBs analogues (+25%). 145 young sedentary adults were assigned to a control (CON, n = 54), a MOD-EX (n = 48) or a VIG-EX (n = 43). 102 participants were included in the final long-term analyses (CON, n = 36; MOD-EX, n = 33; and VIG-EX, n = 33) of the trial. After 24-week of supervised exercise, MOD-EX decreased plasma levels of omega-6 oxylipins, concretely linoleic acid (LA) and adrenic acid derived oxylipins, and the eCBs analogues OEA and LEA in comparison to the CON (all P ≤ 0.021). VIG-EX decreased LA-derived oxylipins and LEA compared to CON. No relevant adverse events were recorded.

**Interpretation:**

Endurance and resistance exercises acutely increased plasma levels of oxylipins, eCBs, and their analogues, whereas 24 weeks of exercise training decreased fasting plasma levels of omega-6 oxylipins, and eCBs analogues in young, sedentary adults.

**Funding:**

See Acknowledgments section.

**Research in Context**

**Evidence before this study**

Regular exercise decreases the risk of mortality and the prevalence of chronic diseases partially by the modulation of the inflammatory and immune status. Recently, fatty acid-derived lipid mediators, including oxylipins, endocannabinoids (eCBs), and their analogues have emerged as metabolites involved in the inflammatory and immune response to physiological stressors. Endurance exercise acutely increases circulating levels of oxylipins and eCBs. However, the acute effects of resistance exercise or the long-term effects of exercise training on these lipid mediators remain to be ascertained.

**Added value of this study**

Here, we report a comprehensive overview of exercise's acute and long-term effects on plasma levels of oxylipins, eCBs, and their analogues in young, sedentary adults. We show that acute endurance and resistance exercise increase plasma levels of arachidonic acid (AA) omega-6 derived oxylipins, eicosapentaenoic acid and docosahexaenoic acid omega-3 derived oxylipins, as well as anandamide and eCBs analogues in young adults. In addition, a 24-week supervised concurrent exercise intervention-based randomized controlled trial was carried out. We reveal that after 24 weeks of exercise training, plasma levels of omega-6 oxylipins and eCBs analogues are decreased compared to a control group.

**Implications of all the available evidence**

Our findings describe, the effects of both acute endurance and resistance exercise and long-term concurrent exercise intervention on levels of oxylipins, eCBs, and their analogues. The acute and long-term effects of exercise on the lipid mediators observed in the present study are comparable to those on other inflammatory mediators (i.e., interleukin-6). Therefore, our results suggest that these metabolites could be key mediators of the inflammatory and immune response to exercise. These findings also provide novel insights into the health-related benefits of exercise, specifically by the modulation of the metabolic pathways of novel lipid mediators such as oxylipins and eCBs.

## Introduction

Regular exercise is related to lower odds of mortality and lower prevalence of chronic diseases due to its benefits on cardiovascular, metabolic, and immune health.^1^^,^^2^ Although the physiological mechanisms of exercise are multifactorial, the health-related benefits of exercise may be partially explained by modulation of the inflammatory and immune status.^2^^,^^3^ Acute exercise exerts a pro-inflammatory stimulus, whereas long-term exercise training induces anti-inflammatory effects implicated in the health-related benefits of exercise training.^2^^,^^3^ The physiological mechanisms that underlie these effects have primarily focused on muscle-derived myokines, among which interleukin-6 (IL-6), but may also involve additional metabolic tissues and metabolites. Therefore, future research is needed to fully understand the metabolic pathways through which exercise improves inflammation and immune function.^2^^,^^4^

Recently, novel lipid mediators, such as oxylipins and endocannabinoids (eCBs), have emerged as key metabolites in the inflammatory and immune response of an organism to infections and injuries.^5^, ^6^, ^7^ Oxylipins are oxidation products of polyunsaturated fatty acids (PUFAs), with either pro-inflammatory or immune impairing functions (i.e., omega-6 derived oxylipins) or anti-inflammatory, pro-resolution and improved immune functions (i.e., omega-3 derived oxylipins).^5^, ^8^, ^9^ They are the main mediators of the effects of PUFAs on metabolism in humans through their binding to G protein-coupled receptors (GPCRs) or peroxisome proliferator-activate receptors (PPARs).^8^ eCBs, mainly represented by anandamide (AEA) and 2-arachidonyl glycerol (2-AG), activate the G protein-coupled cannabinoid receptors type 1 (CB1R) and 2 (CB2R).^10^^,^^11^ Structural analogues of eCBs, such as palmitoylethanolamide (PEA) and oleoylethanolamide (OEA), have significantly less affinity for CB1R and CB2R,^10^ but have higher affinity for GPCRs, PPARs or transient receptor potential vanilloid (TRPV).^10^ eCBs and their analogues are implicated in the maintenance of the inflammatory and immune status through the modulation of cytokine production and function of immune cells.^10^, ^11^, ^12^

Previous studies in humans have reported that endurance exercise acutely increases circulating levels of oxylipins and eCBs 120–180 min after exercise.^13^, ^14^, ^15^, ^16^, ^17^ The increase in circulating oxylipins is generally more minor after low-exercise intensity or very short exercise than more intense or prolonged exercise bouts.^13^, ^14^, ^15^, ^16^, ^17^ Circulating levels of AEA^18^, ^19^, ^20^, ^21^, ^22^, ^23^ and of the eCBs analogues PEA and OEA^24^ also increase in response to acute endurance exercise in humans.

The long-term effect of exercise training on these lipid mediators is poorly understood.^3^^,^^13^, ^23^ So far, two independent studies have shown that long-term (14 and 145 days) endurance exercise decreases urinary oxylipins such as isoprostanes and plasma prostaglandins in humans.^25^^,^^26^ On the other hand, we found no evidence of 12 weeks of different training modalities on the plasma levels of oxylipins, eCBs, and their analogues in middle-aged adults.^27^ This lack of effect might be explained by low sample size.

Therefore, this study aims to investigate the acute effects of endurance and resistance exercise on plasma levels of oxylipins, eCBs and their analogues, and to study the effects of a 24-week supervised concurrent exercise intervention at moderate and vigorous intensities on the plasma levels of these metabolites in young, sedentary adults.

## Methods

### Participants

**Fig. 1:**
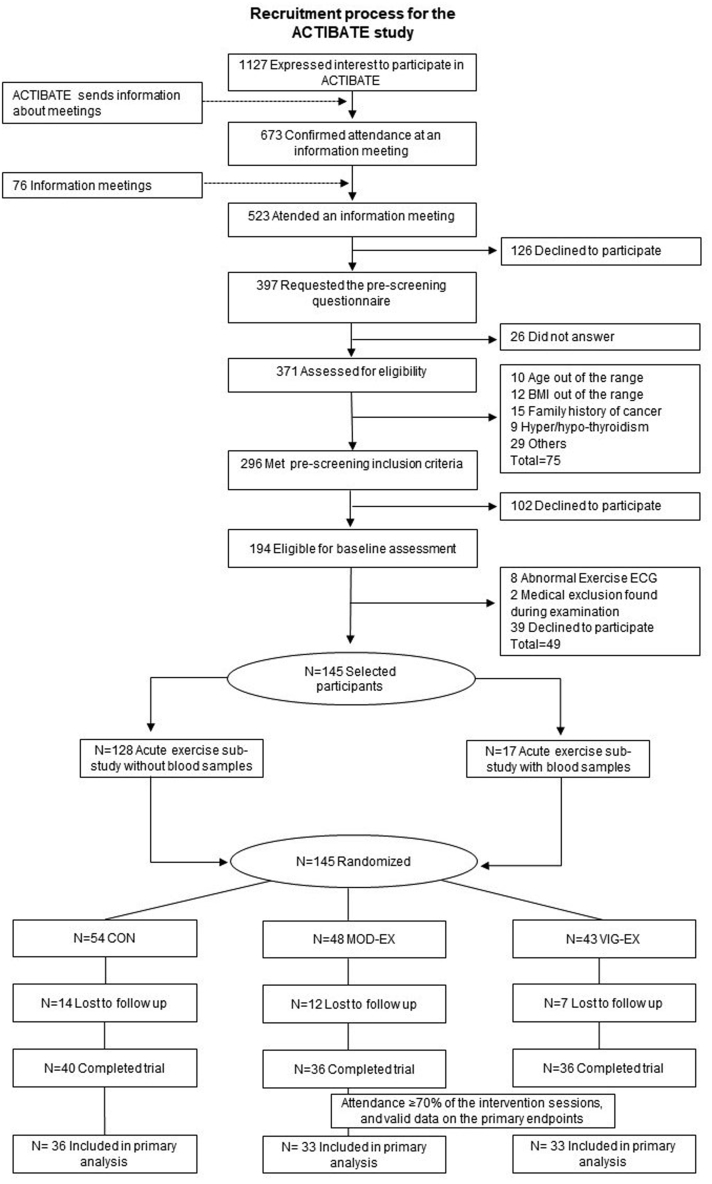
**Study participants enrolment from the ACTIBATE study**. Abbreviations: BMI, body mass index; CON, control group; MOD-EX, moderate-intensity exercise group; VIG-EX: vigorous-intensity exercise group; ECG, electrocardiogram.

A total of 145 young sedentary Caucasian male and female adults between 18 and 25 years old participated in the ACTIBATE study (ACTivating Brown Adipose Tissue through Exercise; ClinicalTrials.gov ID: NCT02365129; Fig. 1).^28^ Participants were recruited through social networks, local media, and posters in Granada (Spain). Inclusion criteria included i) reporting to be sedentary (i.e., <20 min/day of moderate-to-vigorous physical activity for <3 days/week), ii) being non-smoker; and iii) having stable body weight over the last 3 months. Exclusion criteria included diagnostic of diabetes, hypertension, or other significant chronic medical conditions that can interfere with or be aggravated by exercise, being pregnant, using medication deemed to affect energy metabolism, and having frequent exposure to cold temperatures.

### Study design

The current report includes a sub-study and secondary analyses from the single-centre ACTIBATE randomized controlled trial, of which the detailed study design is described elsewhere.^28^ It follows the CONSORT guidelines (Table S1). The ACTIBATE randomized controlled trial showed a lack of effect of exercise on brown adipose tissue (BAT) volume and activity in young sedentary adults. This lack of effect was similarly observed in males and females and in the different areas where BAT is located. On the other hand, exercise reduced adiposity (average −11%), and improved muscular (+15%) and cardiorespiratory fitness (+11%). However, these changes were not correlated with initial BAT volume or activity, nor any changes therein.^29^

**Fig. 2:**
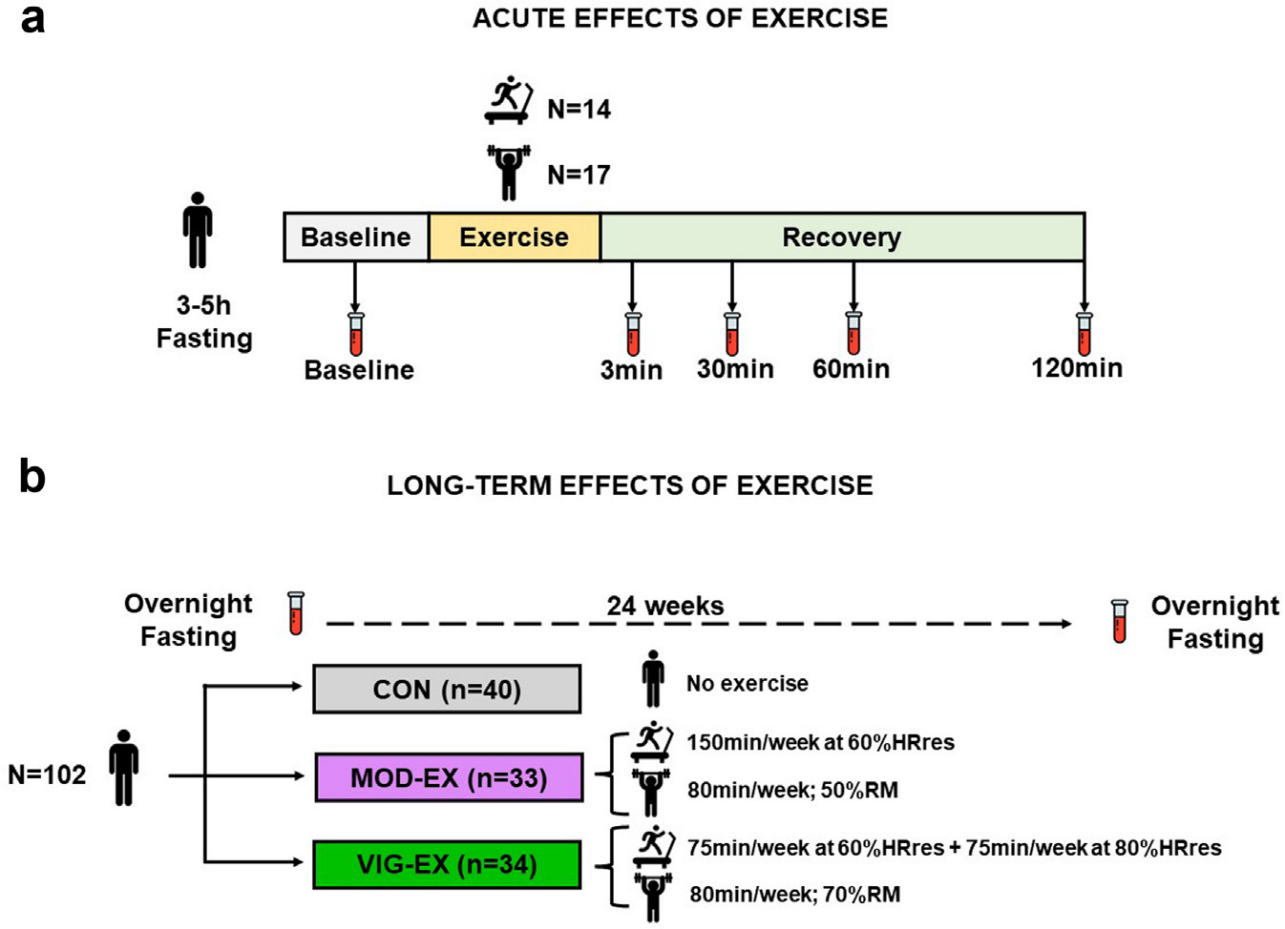
**Design of the study investigating the acute (a) and long-term (b) effects of exercise on plasma levels of oxylipins, eCBs and their analogues in young adults**. Abbreviations: CON, control group; HRes, heart rate reserve; min: minutes: MOD-EX, moderate-intensity exercise group; RM, repetition maximum; VIG-EX, vigorous-intensity exercise group.

During baseline examinations, participants performed an acute and endurance exercise session test. Blood samples were collected in a subgroup of participants who to investigate the acute effects of endurance (n = 14) and resistance (n = 17) exercise on plasma levels of oxylipins, eCBs, and their analogues (acute exercise sub-study; Fig. 2a). After baseline examinations, all participants were randomly assigned into three groups using computer-generated simple (unrestricted) randomization by the principal investigator,^30^ namely, (i) control group (no exercise), (ii) moderate-exercise intensity group (MOD-EX), and (iii) vigorous-exercise intensity group (VIG-EX) (Fig. 2b). The randomization was unblinded and performed by JRR using an in-house system, and no additional researcher had access to it. Participants were explicitly informed of the group to which they were assigned and no delay was experimented between randomization and the initiation of the intervention. Rigorous standardization procedures for data collection and intervention were followed to ensure the internal and external validity of the trial.^31^ The study was conducted over two consecutive years in 4 different waves (from September 2015 to June 2016, and from September 2016 to June 2017) and was ended when the exercise intervention finished. The study was performed in the Sport and Health University Research Institute and the Virgen de las Nieves University Hospital of the University of Granada. All participants were instructed not to change their daily routine, physical activity and dietary patterns throughout the study. No important changes were performed in the methodology or outcomes after the beginning of trial and no relevant adverse events were recorded.

### Acute exercise sessions

The exercise session to examine the acute effect consisted of one endurance session and one resistance exercise session in two independent study days. Both were performed in a fasted state (i.e., 3–5 h fasting), after avoiding stimulants (e.g., caffeine), and any moderate or vigorous exercise the days before the trials (24 h and 48 h, respectively). The endurance exercise trial was a maximum effort test on a treadmill (Pulsar treadmill, H/P/Cosmos Sports & Medical GmbH, Nussdorf-Traunstein, Germany) according to the modified Balke protocol.^32^ The resistance exercise trial was comprised of a maximum isometric strength test in leg press, a handgrip strength test and two 1-repetition maximum (1-RM) tests in bench and leg press, as described elsewhere.^33^ An extensive description of both exercise sessions can be found in the supplementary methods.

### Long-term exercise intervention

An extensive description of the supervised exercise training program can be found elsewhere.^28^ The supervised exercise intervention within the ACTIBATE project combined endurance and resistance training, as the World Health Organization (WHO) guidelines recommended. For 24 weeks, participants attended the research centre 3–4 times per week, 60–90 min per session. Both endurance and resistance training were personalized to the participants’ physical fitness levels. The intervention was divided into 5 phases of different durations, starting with a familiarization phase of 4 weeks.^28^

Participants completed a total of 150 min/week of endurance training (performed in all sessions), performed at 60% of the heart rate reserve (HRres) in the MOD-EX, whereas the VIG-EX performed 75 min/week at 60% HRres and 75 min/week at 80% HRres. The participants completed a total of 80 min of resistance exercise per week (performed over 2 sessions) with loads equivalent to the 50%RM in the MOD-EX and to the 70%RM in the VIG-EX. The load for resistance exercises was individually adjusted monthly.^28^

Exercise sessions were carried out in groups of 10–16 participants, at the same time of the day during the whole intervention. Attendance of the participants was registered. The adherence to the prescribed intensity for the endurance training was quantified by heart rate monitors (RS800CX, Polar Electro Öy, Kempele, Finland). If participants were unable to attend a session at the research centre, they were allowed and encouraged to perform unsupervised training sessions elsewhere.

### Blood sample collection

Prior to each acute exercise session, an intravenous catheter was placed in the antecubital vein and blood was collected before each session and 3, 30, 60 and 120 min after the end of each session. For the evaluation of the long-term effects of exercise, blood samples were collected 1–3 weeks before and 3–4 days after the 24-week exercise intervention, in the morning after a 10 h overnight fast. Blood samples were obtained with EDTA-coated Vacutainer® Hemogard™ tubes and were immediately centrifuged to obtain plasma. Samples were aliquoted and stored at −80 °C.

### Determination of plasma levels of oxylipins, endocannabinoids and their analogues

Plasma levels of oxylipins, eCBs, and their analogues were determined using a validated liquid chromatography-tandem mass spectrometry (LC-MS/MS) method as described elsewhere.^34^ Briefly, plasma samples were prepared with liquid–liquid extraction and analysed using a Shimadzu LC system (Shimadzu Corporation, Kyoto, Japan) connected to a SCIEX QTRAP 6500^+^ mass spectrometer (SCIEX, Framingham, MA, USA). In addition to the study samples, quality control (QC) samples were analysed and used to evaluate the quality of the data and correct for between-batch variations. The relative standard deviations (RSDs) of the peak area ratios were calculated for each analyte present in the QC samples. The supplementary material extensively describes the sample preparation, LC-MS/MS analysis, and data pre-processing.

The LC-MS/MS protocol enabled the determination of oxylipins derived from the omega-6 PUFAs [i.e., linoleic acid (LA), dihomo-γ-linolenic acid (DGLA), arachidonic acid (AA), and adrenic acid (AdrA)], as well as the oxylipins derived from omega-3 PUFAs [i.e., α-linolenic acid (ALA), eicosapentaenoic acid (EPA), and docosahexaenoic acid (DHA)]. The area peak ratio of all oxylipins derived from omega-6 PUFAs (LA, DGLA, AA, AdrA) and omega-3 PUFAs (ALA, EPA, and DHA) covered by the analytical method were summed from the individual data (see Table S2 for the oxylipins included in each sum). Additionally, the ratio of omega-6/omega-3 oxylipins was calculated by dividing the sum of omega-6 oxylipins by the sum of omega-3 oxylipins.

This protocol was also used to determine eCBs [i.e., AEA and 2-AG] and their analogues [i.e., docosahexaenoyl ethanolamide (DHEA), dihomo-gamma-linolenoylethanolamide (DGLEA), linoleylethanolamine (LEA), α-linolenoylethanolamide (α-LEA), PEA, pentadecanoylethanolamide (PDEA), palmitoleoylethanolamide (POEA), OEA, stearoylethanolamine (SEA), 2-linoleoylglycerol (2-LG) and 2-oleoylglycerol (2-OG].

The RSD from QC samples of each analyte are listed in Table S2, whereas the internal standards used are listed in Table S3. In the acute exercise samples, of all the analytes detected, 47 showed a low analytical variability with QC_RSD_ ≤15%, 21 showed a moderate analytical variability QC_RSD_ between 15% and 30%. In the long-term exercise samples 47 metabolites showed a low analytical variability, and 18 showed a moderate analytical variability in the long-term exercise samples. All metabolites had a fair variability with intraclass correlation coefficients higher than 0.6.

### Anthropometric and body composition measurements

Weight and height were measured barefoot and with light clothing, using a SECA scale and stadiometer (model 799; Electronic Column Scale, Hamburg, Germany), and were used to calculate body mass index (BMI; kg/m^2^). Lean mass, fat mass and visceral adipose tissue (VAT) mass were measured by dual-energy X-ray absorptiometry using a Discovery Wi device (Hologic Inc., Bedford, MA, USA) equipped with analysis software (APEX version 4.0.2). Fat mass was also expressed as a percentage of body weight.

### Physical fitness assessment

Physical fitness was assessed during the two sessions described above for the collection of samples to test the acute effects of exercise (all participants underwent a physical fitness evaluation while only 14/17 provided blood samples) (see supplementary material).

### Dietary self-reported intake

Dietary self-reported intake was assessed from 24 h recalls taken on three separate days before and after the trial, as previously described.^35^ Data from the 24 h recalls were introduced in the EvalFINUT® software (FINUT, Spain) and the self-reported intake of PUFAs was obtained.

### Basal fat oxidation and maximal fat oxidation

Basal fat oxidation (BFox) was measured by indirect calorimetry (IC) following current recommendations early in the morning.^36^ Maximal fat oxidation during exercise (MFO) was assessed by IC using an incremental treadmill test, and following a previously validated methodology.^37^ An extensive description of the methodology has been published elsewhere.^37^

### Ethics

The study protocol and experimental design were applied in accordance with the last revised ethical guidelines of the Declaration of Helsinki. The study was approved by the Ethics Committee on Human Research of the University of Granada (no. 924) and the Servicio Andaluz de Salud (Centro de Granada, CEI-Granada); all participants gave informed consent.

### Sample size

This study includes secondary analyses from a randomized controlled trial aimed at determining the effects of a 24-week supervised exercise intervention on BAT, which was originally powered to detect changes in BAT volume and activity. Increases of 10% and 20% were anticipated in activated BAT volume in the MOD-EX and VIG-EX groups respectively (rising from a baseline level of 50–70 mL), along with a standard deviation of 50–60 mL.^29^ Differences of at least 10% in BAT volume could be detected with a power of >80% and an α of 0.05 in a group of 34 participants per study group.^29^ Therefore, assuming a maximum loss to follow-up of 30%, 150 participants were targeted (i.e., 50 per group).^29^ The IBM-SPSS Sample power software (version 22) was used for calculations.^29^ Since the current study is based on a sub-study and secondary analysis from the ACTIBATE randomized controlled trial, no specific power calculation was performed. Participants with blood sample determinations were included in this sub-study and secondary analyses.

### Statistical analyses

Descriptive data are expressed as mean ± standard deviation unless otherwise stated. Firstly, data normality was explored using the Shapiro–Wilk test, visual histograms, and Q–Q plots. None of the oxylipins, eCBs, and their analogues followed a normal distribution. Thereby, all values were log2-transformed for the analyses of the acute effects and log10-transformed for the analyses of the long-term effects.

The Sex × Time interaction effects on oxylipins, eCBs, and their analogues were analyzed using a mixed model, with “sex” and “time” as fixed effects (data not shown). Since no sex interactions were observed in any of the analyses mentioned above (all P > 0.1), all analyses were performed after combining men and women data.

The acute effects of endurance and resistance exercise on plasma levels of oxylipins, eCBs, and their analogues were analysed by repeated measures analysis of variance (ANOVA). The fold changes relative to baseline were calculated with the log2-transformed outcomes (i.e., 120 min fold change = log2 area ratio at 120 min minus log2 area ratio at baseline). To account for multiple testing across changes in levels of oxylipins, eCBs, and their analogues, we used the two-stage step-up method of the Benjamini-Hochberg false discovery rate (FDR; 0.25) method.^38^ Pearson correlation analyses were further performed to evaluate the associations between acute changes in plasma levels of oxylipins, eCBs, and their analogues (i.e., 3- and 120-min fold-changes relative to baseline) and baseline adiposity and physical fitness.

No imputation was conducted on the exercise intervention's long-term effects on plasma levels of oxylipins, eCBs, and their analogues. All analyses were performed following the per protocol approach. First, a delta (Δ = log10 post intervention– log10 baseline values) was calculated for every outcome. Then, mean changes induced by the exercise intervention were analysed using a restricted maximum likelihood (REML)-based repeated measures approach combined with the Newton Raphson Algorithm. Analyses included the fixed, categorical group of intervention, time, and time x group interaction. The Kenward-Roger approximation was used to estimate denominator degrees of freedom. Significance tests were based on least-squares means using a two-sided α = 0.05 (two-sided 95% confidence intervals). Bonferroni posthoc adjustments for multiple comparisons were used to examine differences between groups. Additionally, Pearson correlations were performed to analyze the associations between the Δ of oxylipins, eCBs, and their analogues and the Δ of adiposity and physical fitness parameters, cardiometabolic risk parameters, or dietary self-reported intake (i.e., PUFA intake).

All analyses were performed using the Statistical Package for the Social Sciences v.26.0 (IBM Corporation, Chicago, IL, USA), and figures were built with GraphPad Prism software v.9 (GraphPad Software, San Diego, CA, USA). Statistical significance was set at P < 0.05.

### Role of funding source

Funders did not participate in the study design, data collection, data analyses, interpretation, or manuscript writing.

## Results

Among the initial 145 participants allocated to any of the three groups for the long-term intervention, 102 participants were included in the analysis (Fig. 1). 43 participants were excluded from the main analyses as they did not complete the study (i.e., they attended less than 70% of the total training sessions) or they did not have valid measurements for oxylipins, eCBs, and their analogues (Fig. 1). Phenotypical traits of the participants included in the acute and long-term effects of exercise can be found in Table 1 and baseline levels of oxylipins, eCBs and their analogues in Table S4.

**Table 1:** Baseline characteristics of the study participants.

	Acute effect of exercise (sub-study)				Long-term effect of exercise (secondary analyses)					
	Endurance (n = 14)		Resistance (n = 17)		CON (n = 36)		MOD-EX (n = 33)		VIG-EX (n = 33)	
	Mean	SD	Mean	SD	Mean	SD	Mean	SD	Mean	SD
Demographics										
Age (years)	21.8	2.5	22.4	2.5	22.1	2.1	22.1	2.2	22.2	2.5
Male (n, %)	2	14%	6	35%	16	40%	9	27%	10	30%
Female (n, %)	12	86%	11	64%	24	60%	24	73%	24	70%
Caucasian (n, %)	14	100%	17	100%	36	100%	33	100%	33	100%
Body composition										
BMI (kg/m^2^)	24.2	4.0	25.3	4.2	24.3	4.2	25.1	4.3	25.2	4.4
Lean mass (kg)	39.6	7.2	41.8	9.0	41.9	10.8	40.9	7.9	42.0	9.5
Fat mass (kg)	24.1	9.5	26.2	6.7	23.3	7.5	25.6	9.1	25.8	7.9
Fat mass (%)	35.9	10.0	37.1	6.3	34.5	7.2	36.6	8.6	36.5	6.7
VAT mass (g)	326	173	378	160	323	165	359	184	360	185
Physical Fitness										
Handgrip strength (kg)	30.3	5.1	31.6	9.1	31.9	8.2	30.6	7.7	31.0	7.7
RM leg press (kg)	205.2	54.8	210.3	69.6	207.3	74.7	193.3	57.8	200.5	67.4
RM bench press (kg)	28.0	10.0	31.6	13.1	33.2	17.0	28.6	10.5	30.8	13.0
VO_2_peak (ml/kg/min)	40.7	7.2	40.0	9.6	42.5	9.2	40.5	6.7	40.2	8.7
Time to exhaustion (s)	806	236	872	219	892	231	938	191	949	179

Data are presented as mean and standard deviation (SD) unless otherwise stated.

Abbreviations: BMI, body mass index; CON, control group: MOD-EX, moderate-intensity exercise group; RM, repetition maximum; VAT, visceral adipose tissue; VIG-EX, vigorous-intensity exercise group; VO_2_, oxygen consumption.

### Endurance and resistance exercise acutely alter plasma levels of oxylipins, endocannabinoids, and their analogues

**Fig. 3:**
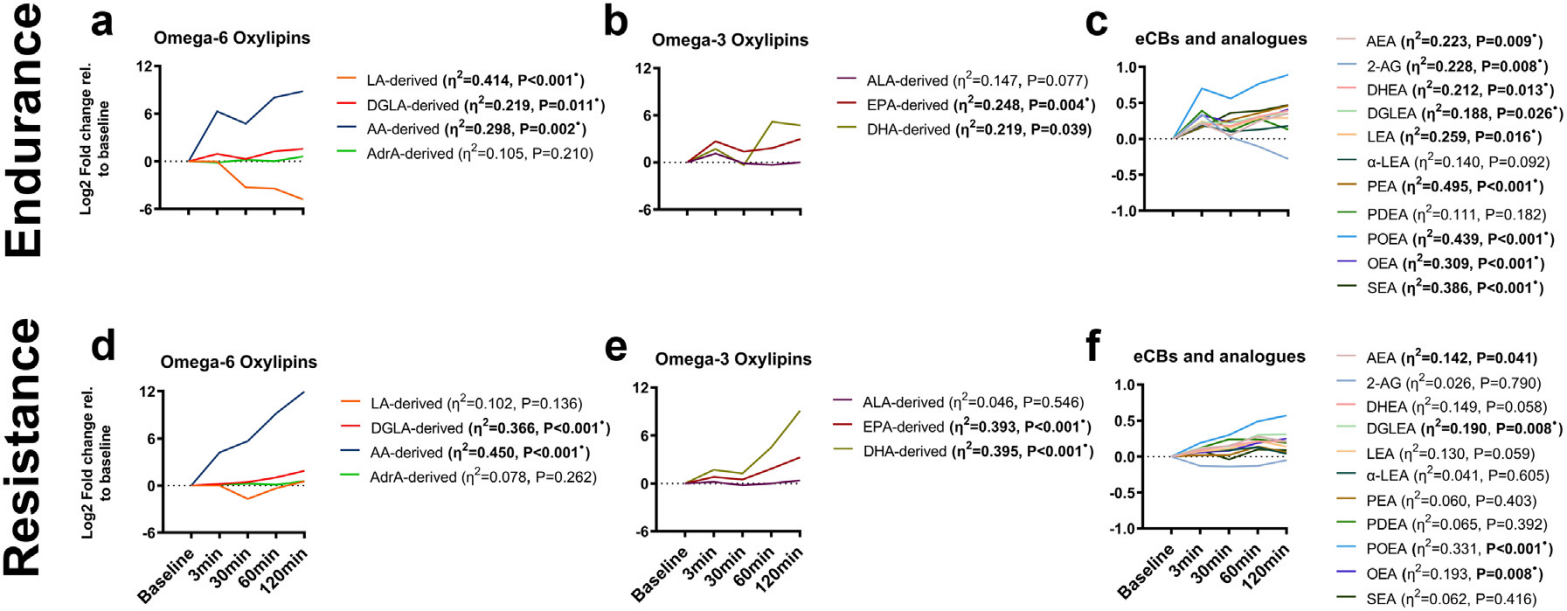
**Endurance and resistance exercise acutely increase plasma levels of oxylipins, endocannabinoids, and their analogues in young adults**. Changes in omega-6 (a, d), omega-3 oxylipins (b, e) and endocannabinoids and their analogues (c, f) after an endurance (a, b, c) and resistance (e, e, f) exercise session. Each line represents the trajectory of the mean log2 fold change relative to baseline of each group of oxylipins or individual endocannabinoid and endocannabinoids analogue. The sum of LA-, DGLA-, AA-, AdrA-, ALA-derived, EPA-, and DHA-derived oxylipins were calculated (a, b, c, d, e). Depicted η^2^ and P values are obtained from repeated measures analyses of variance (ANOVA). • symbol means statistically significant after FDR corrections. Abbreviations: 2-AG, 2-arachidonylglycerol; AA, arachidonic acid; AdrA, adrenic acid; AEA, anandamide; ALA, α-linolenic acid; CON, control group; DGLA, dihomo-γ-linolenic acid; DGLEA, dihomo-gamma-linolenoyl ethanolamide; DHA, Docosahexaenoic acid; DHEA, docosahexaenoyl ethanolamide; ECBs, endocannabinoids; EPA, eicosapentaenoic acid; LA, linoleic acid; LEA, linoleoyl ethanolamide; MOD-EX, moderate intensity exercise group; OEA, oleoyl ethanolamine; PDEA, entadecenoyl ethanolamide; PEA, palmitoyl ethanolamide; POEA, palmitoleoyl ethanolamide; SEA, stearoyl Ethanolamide; VIG-EX, vigorous intensity exercise group; α-LEA, α-Linolenoyl ethanolamide.

Overall, a single session of endurance exercise showed statistically significant increments in the omega-6/omega-3 oxylipins ratio (P = 0.014; Fig. S1a and Table S4). Specifically, endurance exercise displayed statistically significant decrements in LA-derived omega-6 oxylipins (−33.1%; P < 0.001; Figs. 2a and S2a and Table S5), whereas it showed statistically significant increments in DGLA- and AA-derived omega-6 oxylipins (+48.5% and +59.9%, respectively; both P ≤ 0.011; Figs. 2a and S2a and Table S5). Moreover, endurance exercise increased, with statistical significance, EPA-, and DHA-derived omega-3 oxylipins (+88.7% and +56.3%; all P ≤ 0.039; Figs. 3b and S2c and Table S5), without affecting ALA-derived oxylipins. Endurance exercise also acutely increased AEA (+31%; P = 0.009) and decreased 2-AG (−14.6%; P = 0.008) (Fig. 3c and Table S5), as well as their analogues (i.e., DHEA, DGLEA, LEA, PEA, OEA, and SEA; ∼+35%, all P ≤ 0.026; Fig. 3c and Table S5).

In contrast, we found that a single session of resistance exercise did not modify the ratio of omega-6/omega-3 oxylipins (P = 0.886; Fig. S1b and Table S6). However, resistance exercise increased, in a statistically significant manner, DGLA- and AA- derived omega-6 oxylipins (+23.3% and +86.9%; both P < 0.001; Figs. 3d and S2b and Table S6), as well as EPA- and DHA-derived omega-3 oxylipins (+52.0% and +31.4%; both P < 0.001; Figs. 3e and S2d, and Table S6). Resistance exercise also acutely increased AEA, DGLEA, POEA, and OEA (∼+13%, all P ≤ 0.041; Fig. 3f and Table S6).

Overall, all statistically significant results persisted after adjusting for false discovery rate, except for the changes in DHA-derived oxylipins after the endurance exercise session and changes in AEA after resistance exercise.

### Long-term exercise training decreases plasma levels of omega-6 oxylipins

**Fig. 4:**
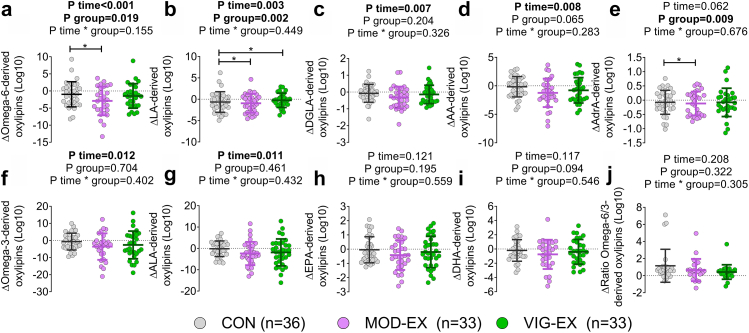
**Long-term moderate, but not vigorous, intensity exercise training decreases plasma levels of omega-6-derived oxylipins in young adults**. a–j: For the analyses, the sum of omega-6 (a), LA-derived (b), DGLA-derived (c), AA-derived (d), AdrA-derived (e), omega-3 (f), ALA-derived (g), EPA-derived (h), DHA-derived (i), and the ratio of omega-6/omega-3 (M) oxylipins were calculated. Δ was calculated as post minus baseline values of the area peak ratio for each oxylipin group. P values obtained from linear mixed repeated measures analyses. Data represent mean and standard deviation. ∗ Symbol means statistically significant differences between groups after post-hoc Bonferroni correction. Abbreviations: CON, control group; ECBs, endocannabinoids; MOD-EX, moderate-intensity exercise group; VIG-EX, vigorous-intensity exercise group; AA, arachidonic acid; AdrA, adrenic acid; ALA, α-linolenic acid; CON, control group; DGLA, dihomo-γ-linolenic acid; DHA, Docosahexaenoic acid; EPA, eicosapentaenoic acid; LA, linoleic acid; MOD-EX, moderate-intensity exercise group; VIG-EX, vigorous-intensity exercise group.

After 24-weeks of exercise intervention MOD-EX decreased, in a statistically significant manner, omega-6-derived oxylipins (−14.9%), compared to CON (95% CI, −2.758 to −0.214; P = 0.016; Fig. 4a and Table 2). Specifically, MOD-EX displayed a statistically significant decrement in LA- and AdrA-derived oxylipins compared to control group (95% CI, −1.398 to −0.093 and −0.311 to −0.036; P = 0.019 and P = 0.008 respectively; Fig. 4b, and Table 2). In addition, MOD-EX tended to decrease AA--derived oxylipins (−11.7%), compared to CON (95% CI, −1.497 to 0.093; P = 0.103; Fig. 4d, and Table 2), and decreasing with statistical significance PGE2 and 15-HETE compared to CON (95% CI, −0.252 to −0.004, and −0.110 to −0.007; P = 0.041 and P = 0.021 respectively; Table S7).

**Table 2:** Differences in plasma levels of oxylipins, endocannabinoids and their analogues between groups after 24 weeks of exercise intervention.

	MOD-EX vs. CON				VIG-EX vs. CON				MOD-EX vs. VIG-EX			
	Mean difference	SE	95% CI	P	Mean difference	SE	95% CI	P	Mean difference	SE	95% CI	P
Oxylipins												
Omega-6-derived oxylipins	−1.486	0.527	[−2.758; −0.214]	**0.016**	−0.832	0.526	[−2.102; 0.438]	0.345	−0.653	0.548	[−1.978; 0.671]	0.705
LA-derived oxylipins	−0.745	0.270	[−1.398; −0.093]	**0.019**	−0.872	0.270	[−1.525; −0.220]	**0.004**	0.127	0.278	[−0.545; 0.799]	1.000
DGLA-derived oxylipins	−0.128	0.082	[−0.327; 0.071]	0.365	0.004	0.082	[−0.194; 0.203]	1.000	−0.133	0.085	[−0.338; 0.073]	0.361
AA-derived oxylipins	−0.702	0.329	[−1.497; 0.093]	0.103	−0.029	0.328	[−0.820; 0.763]	1.000	−0.673	0.341	[−1.497; 0.151]	0.150
AdrA-derived oxylipins	−0.173	0.057	[−0.311; −0.036]	**0.008**	−0.116	0.057	[−0.253; 0.021]	0.129	−0.057	0.059	[−0.199; 0.084]	0.988
Omega-3-derived oxylipins	−0.708	1.067	[−3.285; 1.869]	1.000	0.150	1.052	[−2.393; 2.693]	1.000	−0.858	1.094	[−3.501; 1.785]	1.000
ALA-derived oxylipins	−0.943	0.757	[−2.770; 0.885]	0.643	−0.416	0.757	[−2.243; 1.412]	1.000	−0.527	0.779	[−2.407; 1.353]	1.000
EPA-derived oxylipins	−0.097	0.174	[−0.518; 0.324]	1.000	0.220	0.174	[−0.199; 0.639]	0.620	−0.317	0.180	[−0.750; 0.117]	0.237
DHA-derived oxylipins	−0.254	0.318	[−1.022; 0.515]	1.000	0.452	0.315	[−0.310; 1.214]	0.460	−0.706	0.327	[−1.497; 0.085]	0.097
Ratio omega-6/3-derived oxylipins	−3.914	3.000	[−11.229; 3.401]	0.586	0.226	2.972	[−7.022; 7.474]	1.000	−4.140	3.090	[−11.675; 3.395]	0.551
Endocannabinoids and their analogues												
AEA	−0.033	0.025	[−0.093; 0.027]	0.561	−0.021	0.025	[−0.082; 0.039]	1.000	−0.012	0.026	[−0.073; 0.050]	1.000
2-AG	0.008	0.078	[−0.180; 0.195]	1.000	0.136	0.077	[−0.051; 0.324]	0.239	−0.129	0.081	[−0.324; 0.066]	0.335
2-LG	−0.034	0.113	[−0.306; 0.238]	1.000	0.055	0.113	[−0.217; 0.327]	1.000	−0.089	0.116	[−0.370; 0.192]	1.000
2-OG	0.012	0.113	[−0.261; 0.286]	1.000	0.119	0.114	[−0.155; 0.394]	0.887	−0.107	0.118	[−0.391; 0.177]	1.000
DHEA	−0.057	0.031	[−0.132; 0.019]	0.214	−0.003	0.031	[−0.079; 0.073]	1.000	−0.054	0.032	[−0.132; 0.024]	0.294
DGLEA	−0.012	0.030	[−0.085; 0.062]	1.000	−0.011	0.030	[−0.084; 0.062]	1.000	−0.001	0.031	[−0.077; 0.075]	1.000
LEA	−0.063	0.023	[−0.117; −0.008]	**0.017**	−0.071	0.023	[−0.126; −0.017]	**0.006**	0.008	0.023	[−0.048; 0.065]	1.000
α-LEA	−0.037	0.025	[−0.098; 0.024]	0.428	−0.021	0.025	[−0.082; 0.040]	1.000	−0.016	0.026	[−0.079; 0.047]	1.000
PEA	−0.018	0.010	[−0.042; 0.007]	0.249	−0.009	0.010	[−0.033; 0.015]	1.000	−0.008	0.010	[−0.033; 0.017]	1.000
PDEA	−0.032	0.022	[−0.085; 0.020]	0.407	−0.014	0.022	[−0.067; 0.038]	1.000	−0.018	0.022	[−0.072; 0.036]	1.000
POEA	−0.061	0.044	[−0.167; 0.045]	0.493	0.008	0.044	[−0.098; 0.113]	1.000	−0.069	0.045	[−0.178; 0.040]	0.391
OEA	−0.054	0.020	[−0.102; −0.006]	**0.021**	−0.023	0.020	[−0.070; 0.025]	0.765	−0.031	0.020	[−0.070; 0.025]	0.374
SEA	−0.021	0.011	[−0.048; 0.007]	0.211	−0.002	0.011	[−0.030; 0.026]	1.000	−0.019	0.012	[−0.047; 0.010]	0.338

Data is presented as estimated mean difference between groups, standard error (SE) and 95% confidence interval (CI) in each time point. P values obtained from post-hoc Bonferroni corrections from the linear mixed repeated measures analyses. Significant p values are highlighted in bold.

Abbreviations: 2-AG, 2-arachidonylglycerol; α-LEA, α-Linolenoyl ethanolamide; AA, arachidonic acid; AdrA, adrenic acid; AEA, anandamide; ALA, α-linolenic acid; CON, control group; DGLA, dihomo-γ-linolenic acid; DGLEA, dihomo-gamma-linolenoyl ethanolamide; DHA, Docosahexaenoic acid; DHEA, docosahexaenoyl ethanolamide; EPA, eicosapentaenoic acid; LA, linoleic acid; LEA, linoleoyl ethanolamide; MOD-EX, moderate-intensity exercise group; OEA, oleoyl ethanolamine; PDEA, pentadecanoyl ethanolamide; PEA, palmitoyl ethanolamide; POEA, palmitoleoyl ethanolamide; SEA, stearoyl Ethanolamide; VIG-EX, vigorous-intensity exercise group.

On the other hand, VIG-EX showed a statistically significant decrement in LA-derived oxylipins compared to CON (95% CI, −1.525 to −0.220; P = 0.004; Fig. 4b and Table 2). Specifically, VIG-EX decreased, in a statistically significant manner, 9,10-EpOME, 9,10-DiHOME, 12,13-EpOME and 12,13-DiHOME (95% CI, −0.190 to −0.017, −0.278 to −0.041, −0.221 to −0.017, and −0.155 to −0.002; all P ≤ 0.044; Table S7). We did not observe changes in omega-3-, ALA-, EPA-, DHA-derived oxylipins or the ratio of omega-6/omega-3 oxylipins after 24 weeks of intervention (all P > 0.05; Fig. 4f–j, and Tables 2 and S7.

**Fig. 5:**
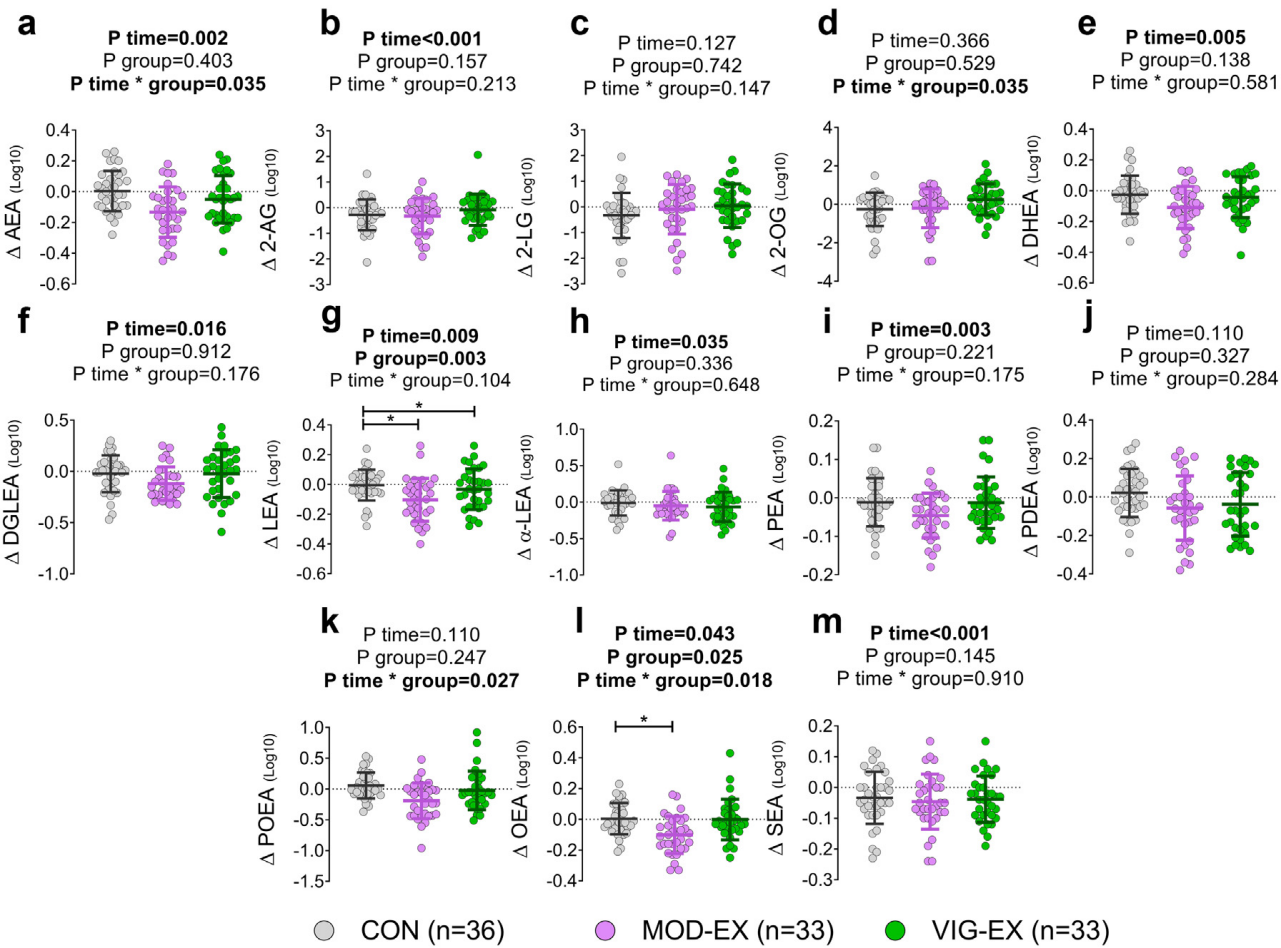
**Long-term moderate, exercise training decreases plasma levels of endocannabinoids analogues in young adults**. a–m: Δ was calculated as post minus baseline values of the area peak ratio for each endocannabinoid and analogue. P value obtained from linear mixed repeated measures analyses. Data represent mean and standard deviation. ∗ Symbol means statistically significant differences between groups after posthoc Bonferroni correction. Abbreviations: 2-AG, 2-arachidonylglycerol; 2-LG, 2-linoleoylglycerol; 2-OG, 2-oleoylglycerol; AEA, anandamide; CON, control group; DGLEA, dihomo-gamma-linolenoyl ethanolamide; DHEA, docosahexaenoyl ethanolamide; LEA, linoleoyl ethanolamide; MOD-EX, moderate-intensity exercise group; OEA, oleoyl ethanolamine; PDEA, pentadecanoyl ethanolamide; PEA, palmitoyl ethanolamide; POEA, palmitoleoyl ethanolamide; SEA, stearoyl ethanolamide; VIG-EX, vigorous-intensity exercise group; α-LEA, α-linolenoyl ethanolamide.

One of the main degradation products of AEA and 2-AG is AA, levels of which tended to decrease in response to MOD-EX. Besides, we found that MOD-EX displayed a statistically significant decrement in LEA (−22.5%), and OEA levels (−20.9%) compared to CON (95% CI −0.117 to −0.008, and −0.102 to −0.006; P = 0.017 and P = 0.021 respectively; Fig. 5g and i and Table 2). On the other hand, VIG-EX decreased LEA levels in a statistically significant manner compared to CON (95% CI, −0.126 to −0.017, P = 0.006; Fig. 5g and Table 2). Although MOD-EX tended to decrease levels of AEA, DHEA, PDEA, or POEA, these changes were not statistically significant compared to CON (Fig. 5 and Table 2).

Overall, all statistically significant results persisted after adjusting for both, baseline values and changes in PUFA intake (Table S8).

### Exercise-induced acute changes in plasma levels of oxylipins and endocannabinoids are related to a better exercise capacity

120 min after acute endurance exercise, we found that changes in AdrA-, ALA- and EPA- derived oxylipins were positively correlated with the time to exhaustion (all r ≥ 0.539, P ≤ 0.047; Fig. S3c). Similarly, changes in eCBs, and their analogues were positively correlated with VO_2peak_ relative to body weight (i.e., AEA, LEA, α-LEA, POEA, OEA; all r ≥ 0.554, P < 0.05; Fig. S3d). 120 min after the bout of resistance exercise, the changes in 2-AG were positively correlated with adiposity (r = 0.786, P < 0.001; Fig. S3h) and negatively correlated with handgrip strength as well as RMs leg press and bench press (all r ≤ −0.495, P ≤ 0.043; Fig. S3h). However, changes in oxylipins, eCBs, and their analogues 3-min and 120-min after acute endurance and resistance exercise were not correlated with BFox and MFO (Table S9).

On the other hand, none of the changes observed on oxylipins, eCBs and their analogues induced by MOD-EX training were related to changes in adiposity, physical fitness capacity, cardiometabolic risk parameters, or dietary self-reported PUFA intake (data not shown). Changes induced by MOD-EX training in AA-derived oxylipins were negatively correlated with changes in MFO (r = −0.520; P = 0.009; Table S10).

## Discussion

Here, we provide an overview of the acute and long-term effects of exercise on plasma levels of oxylipins, eCBs, and their analogues in young, sedentary adults. We found that endurance and resistance exercise acutely increase plasma levels of oxylipins derived from omega-6 and omega-3, as well as of eCBs and their analogues. In contrast, 24 weeks of MOD-EX training, reduced plasma levels of omega-6 oxylipins, as well as the eCBs analogues LEA and OEA; whereas VIG-EX reduced LA-derived omega-6 oxylipins and, the eCB analogue LEA. However, these decreases were not related to the observed reduction in adiposity or improvement in cardiorespiratory fitness. Our results suggest that these metabolites could be key mediators of the inflammatory and immune response to exercise.

The acute and long-term effects of exercise on oxylipins, eCBs, and their analogues observed in the present study are comparable to the effects on other inflammatory mediators. For instance, exercise is an acute pro-inflammatory stimulus that rapidly increases plasma levels of inflammatory molecules such as IL-6.^39^ However, 120–180 min after exercise, the production of anti-inflammatory molecules, such as IL-10, dampening the pro-inflammatory response is increased, favouring the resolution of inflammation.^39^, ^40^, ^41^ When exercise is repeated over time, such in a training program, there is a physiological adaptation leading to a reduced basal pro-inflammatory status (e.g., decrease of basal plasma levels of IL-6.^42^^,^^43^ Therefore, exercise training might modulate plasma levels of oxylipins, eCBs and their analogues similarly to other inflammatory molecules (i.e., IL-6, IL-10).

### Role of oxylipins, endocannabinoids, and their analogues in the acute response to exercise

We unveiled that endurance and resistance exercise acutely increase the plasma levels of AA-, EPA-, and DHA-derived oxylipins in young sedentary adults. This is supported by previous studies that demonstrated that acute endurance exercise increases circulating levels of both omega-6 and omega-3 oxylipins (∼2- to 8-fold).^13^ Exercise induces muscle damage accompanied by increased muscle inflammation and oxidative stress.^7^ Immediately after exercise (3 min in our results), eicosanoids (i.e., prostaglandins, leukotrienes, and lipoxins), which are AA-derived oxylipins, are released to the systemic circulation as a pro-inflammatory response.^5^, ^7^^,^^9^ In this scenario, there is a rapid mobilisation of neutrophils, which migrate from the bloodstream to the damaged tissue,^7^ a process facilitated by AA-derived oxylipins, as we showed in our results, through the induction of vasodilation, vascular permeability, and chemotaxis of neutrophils.^7^ At a later stage during exercise recovery (120 min in our results), a set of anti-inflammatory and pro-resolution oxylipins such as EPA- and DHA-derived oxylipins, which are precursors of the E-series and D-series resolvins, protectins, and maresins, are released to the systemic circulation^5^, ^7^^,^^9^ as we observed in our results. These anti-inflammatory and pro-resolution lipid mediators cease the infiltration of neutrophils, and recruit monocytes that eliminate neutrophils.^7^ Therefore, omega-3 lipid mediators act as ‘stop signals’ to return to a non-inflammatory status after acute exercise and finally control the inflammatory and immune response to muscle damage.^5^, ^7^^,^^9^ Beyond the regulation of inflammation, these changes in plasma levels of oxylipins suggest a complex interplay between different tissues (i.e., skeletal muscle, adipose tissue). Yet, it remains unknown which tissues are the main contributors to the systemic levels of these metabolites.^8^^,^^44^

Here, we demonstrate that eCBs and their analogues acutely increase after a maximal effort (endurance) test and after resistance exercise, suggesting that AEA levels can also be modified at high exercise intensities and by different types of exercise. On the other hand, previous studies have shown that the acute increase in circulating AEA after exercise is dependent on the intensity of exercise, increasing only after moderate-intensity exercise, but not vigorous-intensity exercise.^18^^,^^20^ We observed that changes in eCBs and their analogues were positively correlated with VO_2peak_ relative to body weight, suggesting that the higher the increase of plasma levels of these molecules after exercise, the higher the increase of plasma levels of these molecules was after exercise cardiopulmonary capacity of the individual. Since cardiorespiratory fitness is a well-recognized marker of health status,^45^ these changes also reflect that a higher increase in eCBs and their analogues after exercise are linked to the health status of the individuals.

Akin to oxylipins, changes in eCBs and their analogues could be driven by an interplay between different cells (e.g., myocytes, neurons, adipocytes, or immune cells).^10^^,^^11^ Indeed, eCBs play a role in the modulation of inflammation and the immune system, through their binding to CB2R which is expressed in human leukocytes.^46^ In this sense, eCBs exert both pro-inflammatory and anti-inflammatory or pro-resolution effects which might be crucial in the resolution of inflammation in response to exercise.^46^ Another interesting role attributed to eCBs and their analogues is the modulation of the exercise analgesia effect or the so-called *runner's high*, but further studies are needed to investigate whether a causality exists underlying this relationship.^11^^,^^18^^,^^47^

### Role of oxylipins, endocannabinoids and their analogues in the long-term response to exercise training

High omega-6 oxylipins and eCBs are indicators of higher pro-inflammatory status, high adiposity levels, cardiovascular disruption, liver dysfunction, and/or oxidative stress,^5^, ^7^^,^^9^^,^^10^^,^^12^^,^^46^, ^48^^,^^49^ which concur with the findings observed in our cohort of young, sedentary adults.^50^ Interestingly, we found that long-term exercise training, was an effective therapy to decrease the plasma levels of these metabolites. Acute exercise has been demonstrated to induce higher analgesic effects through increased eCBs levels.^18^^,^^20^ Therefore, we are tempted to speculate that an adaptative response could arise if acute exercise is repeated over time, decreasing the basal levels of omega-6 oxylipins, eCBs, and their analogues. Our results also show that long-term changes in the plasma levels of these metabolites are not related to changes in body composition (i.e., adiposity and lean mass), physical fitness, cardiometabolic risk parameters or self-reported dietary PUFA intake. Interestingly, changes in AA-derived oxylipins were negatively correlated with changes in MFO. A decrease in pro-inflammatory oxylipins, such as AA-derived oxylipins, could induce alterations in genes involved in the regulation of fat oxidation in adipose, liver, and skeletal muscle tissues,^51^ and improve insulin sensitivity in these tissues,^52^ enhancing fat oxidation during exercise. However, the biological implications of the effect of exercise on the plasma levels of oxylipins, eCBs, and their analogues in humans require further investigation. Future studies are needed to elucidate whether a cross-talk between different tissues (e.g., skeletal muscle, adipose tissue, the gut) exists that might be responsible for the changes in the concentrations of these circulating metabolites. Furthermore, it is crucial to be aware that oxylipins and eCBs share common precursors (i.e., PUFAs) and metabolic enzymes (i.e., LOX and CYP).^8^^,^^49^^,^^53^ Intriguingly, we found that MOD-EX tended to decrease AA-derived oxylipins that are also the end products of eCB catabolism. This finding suggests that AA precursors and derived products might play a crucial role during exercise training and should be further investigated.^54^

### Strengths and limitations

The current study shows a number of strengths and limitations. A major strength is the metabolomics-based methodology, targeting all omega-6 and omega-3 oxylipins, eCBs and their analogues. In addition, we have analysed both the acute and long-term responses to exercise, and acute sessions of both endurance and resistance exercises. Nevertheless, a study limitation is the inclusion of only sedentary young adults, which does not allow extrapolation of the findings to older, children, unhealthy, or trained/active populations, and the relatively low sample size of males in the acute experiments. The generalisability of the findings might be dampened since we only include participants who fully completed the exercise intervention in the analyses, and a single trainer primarily executed the exercise training sessions. Due to the low volume of samples, we had to report area peak ratio as proxy of concentration of each metabolite following the Metabolomic Standard Initiative.^55^ Moreover, the long-term effects of exercise were based on a concurrent intervention, without the isolation of long-term effects of aerobic and resistance training. Lastly, the sex heterogeneity of our cohort does not allow to know if the effects of exercise could be sex-dependent.

## Conclusions

This study showed that both endurance and resistance exercise acutely increase plasma levels of oxylipins, eCBs, and their analogues, whereas 24 weeks of exercise training decreases plasma levels of omega-6 oxylipins and eCBs analogues in young sedentary adults. Our findings suggest that both acute and long-term effects of exercise on oxylipins and eCBs might be related to the inflammatory responses to exercise. Further studies are needed to understand the role of exercise in the modulation of the levels of these metabolites and the mechanisms behind exercise benefits on inflammation.

## Contributors

Conceptualization, L.J.-F., I.K., G.S.-D., J.R.R., and B.M.-T.; Methodology, L.J.-F., G.S.-D., F.J.O.-P., L.O.-A., C.M.A., J.M.L.-E., J.R.R. and B.M.-T.; Validation, X.D., W.Y., E.K., A.C.H., T.H., M.S., I.K., P.C.N.R., C.M.A. and J.M.L.-E.; Formal Analysis, L.J.-F., G.S.-D., and B.M.-T.; Data collection, G.S.-D., X.D., W.Y., F.J.O.-P., L.O.-A., E.K., A.C.H., T.H., and I.K.; Data Curation, L.J.-F. and B.M.-T.; Writing – Original Draft, L.J.-F., X.D. and B.M.-T.; Writing – Review & Editing, all authors; Supervision, J.R.R. and B.M.-T.; L.J.-F. and X.D. share first authorship. J.R.R. and B.M.-T. share senior authorship. L.J.-F. and B.M.-T. have verified the underlying data. The order in which they are listed reflects the workload from initiation of the study until publication. All authors commented on the manuscript and approved the final version of the manuscript.

## Data sharing statement

The data that support the findings of this study are available from the corresponding author upon reasonable request, as the study consists of a high number of participants and outcomes and requires specific knowledge for data interpretation.

## Declaration of interests

None.

## Appendix A Supplementary data

Supplemental Tables S1–S10 and Figures S1–S3

Supplemental Tables S1–S10 and Figures S1–S3 Captions

Study protocol
