# Supplementary material for: Acute and long-term exercise differently modulate plasma levels of oxylipins, endocannabinoids, and their analogues in young sedentary adults: A sub-study and secondary analyses from the ACTIBATE randomized controlled-trial
Source: eBioMedicine. 2022 Oct 27;85:104313. doi: 10.1016/j.ebiom.2022.104313 (PMC9626892; doi:10.1016/j.ebiom.2022.104313)
Supplement: Supplemental Tables S1–S10 and Figures S1–S3 [file mmc1.docx]

**SUPPLEMENTAL MATERIAL**

***Supplemental Methods***

1. *Acute exercise sessions*

Endurance exercise trial

Participants walked at 3 km/h for 1 minute and at 4 km/h for 2 minutes for warming up (0% grade) (1). Then, the test started by walking at 5.3 km/h and 0%. From that moment on, the treadmill inclination was increased by 1% every minute, until exhaustion was reached (1). At this point, participants started a 5-minute recovery walking at 4 km/h and 0% inclination (1). During the whole trial, participants were equipped with a heart rate monitor (Polar RS800CX, Polar Electro Öy, Kempele, Finland), 10 electrodes for electrocardiogram monitoring, and a Hans-Rudolph plastic mask (model 7400, Hans Rudolph Inc., Kansas City, MO, USA) connected to a preVent™ metabolic flow sensor (Medical graphics Corp, St Paul, MN, USA) for respiratory gas exchange analyses using a CPX Ultima CardioO2 gas exchange analysis system (Medical Graphics Corp, St Paul, MN, USA). During the test, respiratory gas exchange (oxygen consumption (VO_2_) and carbon dioxide production)) was recorded and the VO_2_peak was determined as the highest observed VO_2_ value, after excluding obvious artifacts if needed.

Resistance exercise trial

Participants first completed the maximum isometric strength test in leg press. After proper allocation in the leg press machine (A300 Leg Press, Model 2531, Keiser Corporation, Fresno CA, USA), participants performed two 3-second repetitions, 2 minutes apart, for which they were instructed and encouraged to push as hard as they could for the whole duration of the repetition.

Afterwards, participants performed the handgrip strength test by completing two repetitions with each hand, 1 minute apart using a Takei 5401 digital Grip-D hand dynamometer (Takei, Tokyo, Japan) (2). For the handgrip strength test, participants remained in a standing position, with the exercising arm parallel and slightly separated from the trunk. The participants were asked to squeeze the grip gradually and continuously, and as hard as possible. Men executed the test with the grip span of the dynamometer fixed at 5.5 cm, while it was adjusted to the individual’s hand size for women, according to a validated equation (2). The highest strength recorded in each hand was selected and the average between both hands was used for the analyses.

Then, participants performed the leg press 1-RM test in the above-mentioned leg press machine. After performing 1 set of 10 repetitions with a self-selected light weight for warming-up, they were instructed to perform 1 set of 8 repetitions selecting the resistance with which they could perform 15 repetitions as much. Later, after a 1-minute recovery, the resistance load was increased by the study personnel, aiming to set a load with which the participant could perform <10 repetitions, and participants were instructed to do as many repetitions as possible. The participants were instructed to stop exercising after 3-4 repetitions if they felt they could perform more than 10 repetitions with the resistance load. If they performed more than 10 repetitions, they rested for 5 minutes and repeated the test with a higher load. The maximum number of attempts for assessing the RM (in a set of <10 repetitions) was 3. Lastly, participants performed the bench press 1-RM test following the procedure described for the leg press, in a bench within a pneumatic power rack (Power rack, Model 3111, Keiser Corporation, Fresno CA, USA).The 1-RM of both exercises was estimated by the equation previously proposed (3).

1. *Determination of plasma levels of oxylipins and endocannabinoid*
   1. *Sample preparation*

Oxylipins were extracted using liquid-liquid extraction under ice-cool conditions as previously described (4). Briefly, 150 µL of plasma samples were transferred into 1.5-mL Eppendorf tubes and spiked with 5 µL of a solution of butylated hydroxytoluene (0.4 mg/mL) and 10 µL of a solution containing isotopically-labelled internal standards (**Table S2**). Then, 150 µL of a buffer solution composed of 0.2 M citric acid and 0.1 M disodium hydrogen phosphate was added prior to the addition of 1000 µL of a 50:50 v/v mixture of methyl tert-butyl ether and butanol. Samples were mixed for 5 min using a bullet blender (Next Advance Inc., Troy, NY, USA) prior to centrifugation for 10 min at 16,000 g and 4°C. The supernatant (900 µL) was collected and evaporated to dryness using a SpeedVac Vacuum Concentrator (Thermo Fisher Scientific, Waltham, MA, USA) prior to reconstitution in 50 µL of a mixture of methanol:acetonitrile (70:30, v/v). The reconstituted samples were centrifuged (16,000 g, 10 min, 4ºC) prior to collection of 40 µL of the supernatant, which was injected into the LC-MS/MS instrument.

- 1. *Profiling of oxylipins and endocannabinoids using liquid chromatography – tandem mass spectrometry*

Oxylipins and endocannabinoids analysis were performed using a previously validated method (4). Briefly, extracted samples were analysed using a Shimadzu LC system (Shimadzu Corporation, Kyoto, Japan) connected to a SCIEX QTRAP 6500^+^ mass spectrometer (AB Sciex, Framingham, MA, USA). Separation was performed using a BEH C18 column (50 mm × 2.1 mm, 1.7 μm) from Waters Technologies (Mildford, MA, USA) maintained at 40°C. The mobile phase was composed of 0.1% acetic acid in water (A), acetonitrile/0.1% acetic acid in methanol (90:10, v/v, B), and 0.1% acetic acid in isopropanol (C). The flow rate was set at 0.7mL/min, whereas the injection volume was 10 μL preceded by the injection of 20 μL of mobile phase. Ionisation of the compounds was performed using electrospray ionisation in negative mode. Selected Reaction Mode (SRM) was used for MS/MS acquisition. SRM transitions were individually optimised for targeted analytes and respective internal standards using standard solutions.

- 1. *Data quality and data pre-processing*

For each target compound, the ratio between its peak area and the peak area of its respective internal standard was calculated using SCIEX OS-MQ Software and used for further data analysis.

The quality of the data was monitored using regular injection of quality control (QC) samples, consisting of blank plasma samples, within the sequence. QC samples were used to correct for inter-batch variations using the in-house developed mzQuality workflow (available at http://www.mzQuality.nl).(5) Relative standard deviations (RSDs) of peak area ratios were calculated for each targeted analyte detected in the QC samples.

**References**

1. Balke B & Ware RW. An experimental study of physical fitness of Air Force personnel. *United States Armed Forces medical journal* 1959 **10** 675–688.

2. Ruiz-Ruiz J, Mesa JLM, Gutiérrez A, & Castillo MJ. Hand Size Influences Optimal Grip Span in Women but not in Men. *The Journal of Hand Surgery* 2002 . (doi:10.1053/jhsu.2002.34315)

3. Wathen D. Load assignment. In T. R. Baechle (Ed.), Essentials of strength training and conditioning. Champaign,.1994.

4. Zazzo A Di, Yang W, Coassin M, Micera A, Antonini M, Piccinni F, Piano M De, Kohler I, Harms AC, Hankemeier T, Boinini S, & Mashaghi A. Signaling lipids as diagnostic biomarkers for ocular surface cicatrizing conjunctivitis. *Journal of Molecular Medicine* 2020 **98** 751–760. (doi:10.1007/s00109-020-01907-w)

5. Kloet FM Van Der, Bobeldijk I, Verheij ER, & Jellema RH. Analytical error reduction using single point calibration for accurate and precise metabolomic phenotyping. *Journal of Proteome Research* 2009 **8** 5132–5141. (doi:10.1021/pr900499r)

***Supplementary results***

**Table S1**. 2017 CONSORT checklist of information to include when reporting a randomized trial assessing nonpharmacologic treatments (NPTs).

| Section/Topic Item | Checklist item no. | CONSORT item | Extension for NPT trials | Reported on page nº |
| --- | --- | --- | --- | --- |
| Title and abstract |  |  |  |  |
|  | 1a | Identification as a randomized trial in the title |  | 1 |
|  | 1b | Structured summary of trial design, methods, results, and conclusions (for specific guidance see CONSORT for abstracts) | *Refer to CONSORT extension for abstracts for NPT trials* | *3,4* |
| Introduction |  |  |  |  |
| Background and objectives | 2a | Scientific background and explanation of rationale |  | 8,9 |
|  | 2b | Specific objectives or hypotheses |  | 9 |
| Methods |  |  |  |  |
| Trial design | 3a | Description of trial design (such as parallel, factorial) including allocation ratio | When applicable, how care providers were allocated to each trial group | 10 |
|  | 3b | Important changes to methods after trial commencement (such as eligibility criteria), with reasons |  | 11 |
| Participants | 4a | Eligibility criteria for participants | When applicable, eligibility criteria for centers and for *care providers* | 10 |
|  | 4b | Settings and locations where the data were collected |  | 11 |
| Interventions*†* | 5 | The interventions for each group with sufficient details to allow replication, including how and when they were actually administered | Precise details of both the experimental treatment and comparator | 11-13 |
|  | 5a |  | Description of the different components of the interventions and, when applicable, description of the procedure for tailoring the interventions to individual participants. | 11-12 |
|  | 5b |  | Details *of whether and* how the interventions were standardized. | 11-12 |
|  | 5c. |  | Details *of whether and* how adherence of care providers to the protocol was assessed or enhanced | 12 |
|  | 5d |  | *Details of whether and how adherence of participants to interventions was assessed or enhanced* | *12* |
| Outcomes | 6a | Completely defined pre-specified primary and secondary outcome measures, including how and when they were assessed |  | 13-15 |
|  | 6b | Any changes to trial outcomes after the trial commenced, with reasons |  | 11 |
| Sample size | 7a | How sample size was determined | When applicable, details of whether and how the clustering by care providers or centers was addressed | 16 |
|  | 7b | When applicable, explanation of any interim analyses and stopping guidelines |  | N/A |
| Randomization: |  |  |  |  |
| - Sequence generation | 8a | Method used to generate the random allocation sequence |  | 11 |
|  | 8b | Type of randomization; details of any restriction (such as blocking and block size) |  | 11 |
| - Allocation concealment mechanism | 9 | Mechanism used to implement the random allocation sequence (such as sequentially numbered containers), describing any steps taken to conceal the sequence until interventions were assigned |  | 11 |
| - Implementation | 10 | Who generated the random allocation sequence, who enrolled participants, and who assigned participants to interventions |  | 11 |
| Blinding | 11a | If done, who was blinded after assignment to interventions (for example, participants, care providers, those assessing outcomes) and how | ~~Whether or not those administering co-interventions were blinded to group assignment~~  If done, who was blinded after assignment to interventions (e.g., participants, care providers, *those administering co-interventions,* those assessing outcomes) and how | 11 |
|  | 11b | If relevant, description of the similarity of interventions | ~~If blinded, method of blinding and description of the similarity of interventions~~ | Not relevant |
|  | 11c |  | *If blinding was not possible, description of any attempts to limit bias* | 11 |
| Statistical methods | 12a | Statistical methods used to compare groups for primary and secondary outcomes | When applicable, details of whether and how the clustering by care providers or centers was addressed | 16-18 |
|  | 12b | Methods for additional analyses, such as subgroup analyses and adjusted analyses |  | 16-18 |
| Results |  |  |  |  |
| Participant flow (a diagram is strongly recommended) | 13a | For each group, the numbers of participants who were randomly assigned, received intended treatment, and were analyzed for the primary outcome | The number of care providers or centers performing the intervention in each group and the number of patients treated by each care provider or in each center | 19, Figure 1 |
|  | 13b | For each group, losses and exclusions after randomization, together with reasons |  | 19, Figure 1 |
|  | 13c |  | *For each group, the delay between randomization and the initiation of the intervention* | *11* |
|  | new |  | Details of the experimental treatment and comparator as they were implemented | 19 |
| Recruitment | 14a | Dates defining the periods of recruitment and follow-up |  | 11 |
|  | 14b | Why the trial ended or was stopped |  | 11 |
| Baseline data | 15 | A table showing baseline demographic and clinical characteristics for each group | When applicable, a description of care providers (case volume, qualification, expertise, etc.) and centers (volume) in each group. | Table 2 |
| Numbers analyzed | 16 | For each group, number of participants (denominator) included in each analysis and whether the analysis was by original assigned groups |  | Figure 1 |
| Outcomes and estimation | 17a | For each primary and secondary outcome, results for each group, and the estimated effect size and its precision (such as 95% confidence interval) |  | 19-22 |
|  | 17b | For binary outcomes, presentation of both absolute and relative effect sizes is recommended |  | Not binary outcomes |
| Ancillary analyses | 18 | Results of any other analyses performed, including subgroup analyses and adjusted analyses, distinguishing pre-specified from exploratory |  | 19-22 |
| Harms | 19 | All important harms or unintended effects in each group (for specific guidance see CONSORT for harms) |  | 11 |
| **Discussion** |  |  |  |  |
| Limitations | 20 | Trial limitations, addressing sources of potential bias, imprecision, and, if relevant, multiplicity of analyses | In addition, take into account the choice of the comparator, lack of or partial blinding, and unequal expertise of care providers or centers in each group | 27 |
| Generalizability | 21 | Generalizability (external validity, applicability) of the trial findings | Generalizability (external validity) of the trial findings according to the intervention, comparators, patients, and care providers and centers involved in the trial | 27 |
| Interpretation | 22 | Interpretation consistent with results, balancing benefits and harms, and considering other relevant evidence |  | 23-26 |
| Other information |  |  |  |  |
| Registration | 23 | Registration number and name of trial registry |  | 2 |
| Protocol | 24 | Where the full trial protocol can be accessed, if available |  | Ref. 31. |
| Funding | 25 | Sources of funding and other support (such as supply of drugs), role of funders |  | 30 |

| **Table S2.** List of oxylipins, endocannabinoids, and endocannabinoids analogues measured. | | | | | |
| --- | --- | --- | --- | --- | --- |
| Group | Abbreviation | Name (International Union of Pure and Applied Chemistry, IUPAC) | ChEBI ID | RSD in QC  acute samples | RSD in QC long-term samples |
|  | Omega-6 oxylipins | |  |  |  |
| *LA-derived oxylipins* | LA | 9Z,12Z-octadecadienoic acid | 17351 | 18.00 | 10.16 |
|  | 9-HODE | (±)-9-hydroxy-10E,12Z-octadecadienoic acid | 72651 | 4.55 | 7.61 |
|  | 13-HODE | (±)-13-hydroxy-9Z,11E-octadecadienoic acid | 72639 | 4.12 | 7.11 |
|  | 9,10,13-TriHOME | 9S,10S,13S-trihydroxy-11E-octadecenoic acid | 34499 | 15.90 | 15.70 |
|  | 9,12,13-TriHOME | 9S,12S,13S-trihydroxy-10E-octadecenoic acid | 34506 | 17.30 | 6.93 |
|  | 9,10-EpOME | 9,10-epoxy-12Z-octadecenoic acid | 34494 | 11.38 | 7.80 |
|  | 9,10-DiHOME | 9,10-dihydroxy-12Z-octadecenoic acid | 72663 | ND | 7.33 |
|  | 12,13-EpOME | (±)-12(13)-epoxy-9Z-octadecenoic acid | 38229 | 5.90 | 9.56 |
|  | 12,13-DiHOME | 12,13-dihydroxy-9Z-octadecenoic acid | 72665 | 4.78 | 6.74 |
|  | 10-NO2-LA | 10-nitro,9Z,12Z-octadecadienoic acid | 34125 | 16.16 | 13.26 |
|  | GLA | 6Z,9Z,12Z-octadecatrienoic acid | 28661 | 18.18 | ND |
| *DGLA-derived oxylipins* | DGLA | 8Z,11Z,14Z-eicosatrienoic acid | 53486 | 13.82 | 23.92 |
|  | 5-HETrE | 5S-hydroxy-6E,8Z,11Z-eicosatrienoic acid | 88359 | 18.06 | ND |
|  | 8-HETrE | 8S-hydroxy-9E,11Z,14Z-eicosatrienoic acid | 140473 | 16.32 | 22.77 |
|  | 15-HETrE | 15S-hydroxy-8Z,11Z,13E-eicosatrienoic acid | 88348 | 10.36 | 13.55 |
| *AA-derived oxylipins* | AA | 5Z,8Z,11Z,14Z-eicosatetraenoic acid | 15843 | 6.10 | 13.45 |
|  | PGE2 | 9-oxo-11R,15S-dihydroxy-5Z,13E-prostadienoic acid | 15551 | 5.93 | 35.97 |
|  | PGF2α | 9α,11α,15S-trihydroxy-prosta-5Z,13E-dien-1-oic acid | 15553 | 3.20 | ND |
|  | 2,3-dinor-8-iso-PGF2α | 9α,11α,15S-trihydroxy-2,3-dinor-(8β)-prosta-5Z,13E-dien-1-oic acid | NA | 15.33 | ND |
|  | 13,14-dihydro-15-keto-PGF2α | 9α,11α-dihydroxy-15-oxo-prost-5Z-en-1-oic acid | 63976 | 5.90 | ND |
|  | TxB2 | 9S,11,15S-trihydroxy-thromboxa-5Z,13E-dien-1-oic acid | 28728 | 14.33 | 6.95 |
|  | 12-HHTrE | 12S-hydroxy-5Z,8E,10E-heptadecatrienoic acid | 63977 | 16.78 | 8.11 |
|  | 8,12-IPF2α-IV | (12α)-5,9α,11α-trihydroxy-prosta-6E,14Z-dien-1-oic acid | NA | 3.17 | 7.20 |
|  | 5-HETE | 5-hydroxy-6E,8Z,11Z,14Z-eicosatetraenoic acid | 28209 | 6.37 | 10.43 |
|  | 8-HETE | 8-hydroxy-5Z,9E,11Z,14Z-eicosatetraenoic acid | 34486 | 7.08 | ND |
|  | 9-HETE | 9-hydroxy-5Z,7E,11Z,14Z-eicosatetraenoic acid | 72786 | 28.39 | ND |
|  | 11-HETE | 11-hydroxy-5Z,8Z,12E,14Z-eicosatetraenoic acid | 72606 | 29.58 | 10.74 |
|  | 12-HETE | 12-hydroxy-5Z,8Z,10E,14Z-eicosatetraenoic acid | 19138 | 14.27 | 11.41 |
|  | 15-HETE | 15-hydroxy-5Z,8Z,11Z,13E-eicosatetraenoic acid | 64017 | 6.99 | 9.33 |
|  | 20-HETE | 20-hydroxy-5Z,8Z,11Z,14Z-eicosatetraenoic acid | 34306 | 6.94 | 9.35 |
|  | 5,6-DiHETrE | 5,6-dihydroxy-8Z,11Z,14Z-eicosatrienoic acid | 63974 | 5.76 | 9.02 |
|  | 8,9-DiHETrE | 8,9-dihydroxy-5Z,11Z,14Z-eicosatrienoic acid | 63970 | 7.56 | 9.91 |
|  | 11,12-EpETrE | 11,12-epoxy-5Z,8Z,14Z-eicosatrienoic acid | 34130 | 16.16 | ND |
|  | 11,12-DiHETrE | 11,12-dihydroxy-5Z,8Z,14Z-eicosatrienoic acid | 63969 | 5.28 | 8.44 |
|  | 14,15-EpETrE | 14,15-epoxy-5Z,8Z,11Z-eicosatrienoic acid | 34157 | 13.81 | 19.47 |
|  | 14,15-DiHETrE | 14,15-dihydroxy-5Z,8Z,11Z-eicosatrienoic acid | 63966 | 4.48 | 7.1 |
| *AdrA-deriv. oxylipins* | AdrA | 7Z,10Z,13Z,16Z-docosatetraenoic acid | 53487 | 16.2 | 22.09 |
|  | 1a,1b-dihomo-PGF2α | 1a,1b-dihomo-9S,11R,15S-trihydroxy-5Z,13E-prostadienoic acid | NA | 7.10 | 22.14 |
|  | Omega-3 oxylipins | |  |  |  |
| *LA-derived oxylipins* | ALA | 9Z,12Z,15Z-octadecatrienoic acid | 27432 | 10.78 | 9.84 |
|  | 9-HOTrE | 9S-hydroxy-10E,12Z,15Z-octadecatrienoic acid | 80447 | 5.39 | 7.61 |
|  | 12,13-DiHODE | (±)-12,13-dihydroxy-9Z,15Z-octadecadienoic acid | 88461 | 4.24 | 5.80 |
| *EPA-derived oxylipins* | EPA | 5Z,8Z,11Z,14Z,17Z-eicosapentaenoic acid | 28364 | 14.56 | 8.55 |
|  | 5-HEPE | (±)-5-hydroxy-6E,8Z,11Z,14Z,17Z-eicosapentaenoic acid | 72801 | 9.58 | 13.12 |
|  | 12-HEPE | (±)-12-hydroxy-5Z,8Z,10E,14Z,17Z-eicosapentaenoic acid | 72645 | 20.29 | 12.07 |
|  | 15-HEPE | (±)-15-hydroxy-5Z,8Z,11Z,13E,17Z-eicosapentaenoic acid | 72627 | 12.95 | ND |
|  | 14,15-DiHETE | (±)-14,15-dihydroxy-5Z,8Z,11Z,17Z-eicosatetraenoic acid | 88459 | 5.20 | 8.02 |
|  | 17,18-DiHETE | (±)-17,18-dihydroxy-5Z,8Z,11Z,14Z-eicosatetraenoic acid | 88349 | 5.25 | 9.06 |
|  | DPA | 7Z,10Z,13Z,16Z,19Z-docosapentaenoic acid | 61204 | 9.68 | 13.97 |
| *DHA-derived oxylipins* | DHA | 4Z,7Z,10Z,13Z,16Z,19Z-docosahexaenoic acid | 28125 | 6.11 | 9.80 |
|  | 4-HDoHE | (±)-4-hydroxy-5E,7Z,10Z,13Z,16Z,19Z-docosahexaenoic acid | 72624 | 18.37 | 14.25 |
|  | 7-HDoHE | (+/-)-7-hydroxy-4Z,8E,10Z,13Z,16Z,19Z-docosahexaenoic acid | 72623 | 29.33 | ND |
|  | 8-HDoHE | (±)-8-hydroxy-4Z,6E,10Z,13Z,16Z,19Z-docosahexaenoic acid | 72610 | ND | 19.71 |
|  | 10-HDoHE | (+/-)-10-hydroxy-4Z,7Z,11E,13Z,16Z,19Z-docosahexaenoic acid | 72640 | 27.78 | ND |
|  | 11-HDoHE | (±)-11-hydroxy-4Z,7Z,9E,13Z,16Z,19Z-docosahexaenoic acid | 72794 | 24.17 | 17.09 |
|  | 13-HDoHE | (±)-13-hydroxy-4Z,7Z,10Z,14E,16Z,19Z-docosahexaenoic acid | 72608 | 23.01 | 12.28 |
|  | 14-HDoHE | (±)-14-hydroxy-4Z,7Z,10Z,12E,16Z,19Z-docosahexaenoic acid | 72647 | 9.46 | 14.75 |
|  | 16-HDoHE | (±)-16-hydroxy-4Z,7Z,10Z,13Z,17E,19Z-docosahexaenoic acid | 72613 | 14.44 | 15.42 |
|  | 17-HDoHE | (±)-17-hydroxy-4Z,7Z,10Z,13Z,15E,19Z-docosahexaenoic acid | 72637 | 17.922 | 9.05 |
|  | 20-HDoHE | (±)-20-hydroxy-4Z,7Z,10Z,13Z,16Z,18E-docosahexaenoic acid | 72615 | 20.23 | 23.60 |
|  | 19,20-EpDPE | (±)-19(20)-epoxy-4Z,7Z,10Z,13Z,16Z-docosapentaenoic acid | 72653 | 8.17 | 13.45 |
|  | 19,20-DiHDPA | (±)-19,20-dihydroxy-4Z,7Z,10Z,13Z,16Z-docosapentaenoic acid | 72657 | 5.07 | 7.89 |
|  | *Endocannabinoids and their analogues* | |  |  |  |
|  | AEA | N-(5Z,8Z,11Z,14Z-eicosatetraenoyl)-ethanolamine | 2700 | 8.61 | 11.33 |
|  | 2-AG | 2-(5Z,8Z,11Z,14Z-eicosatetraenoyl)-sn-glycerol | 52392 | 12.97 | 16.80 |
|  | 2-LG | 2-(9Z,12Z-Octadecadienoyl)-glycerol | NA | ND | 19.17 |
|  | 2-OG | 2-(9Z-octadecenoyl)-sn-glycerol | 73990 | ND | 27.71 |
|  | DHEA | N-(4Z,7Z,10Z,13Z,16Z,19Z-docosahexaenoyl)-ethanolamine | 85252 | 9.41 | 16.53 |
|  | DGLEA | N-(8Z,11Z,14Z-eicosatrienoyl)-ethanolamine | 34488 | 13.08 | 26.65 |
|  | LEA | N-(9Z,12Z-octadecadienoyl)-ethanolamine | 64032 | 4.20 | 5.3 |
|  | α-LEA | N-(9Z,12Z,15Z-octadecatrienoyl)-ethanolamine | 89605 | 9.4 | 14.9 |
|  | PEA | N-hexadecanoyl-ethanolamine | 71464 | 5.47 | 4.05 |
|  | PDEA | N-(Pentadecanoyl)-ethanolamine | N/A | 21.47 | 30.3 |
|  | POEA | N-(9Z-hexadecenoyl)-ethanolamine | 71465 | 9.38 | 13.5 |
|  | OEA | N-(9Z-octadecenoyl)-ethanolamine | 71466 | 19.00 | 7.70 |
|  | SEA | N-(Octadecanoyl)-ethanolamine | 85299 | 8.25 | 10.62 |

ChEBI, Chemical Entities of Biological Interest; N/A, not available; ND, not detected; QC, quality control; RSD, relative standard error.

**Table S3.** List of internal standards used in the LC/MS method.

| **Abbreviation** | **Name (International Union of Pure and Applied Chemistry, IUPAC)** |
| --- | --- |
| Arachidonic Acid-d8 C20:4-w6-d8 | 5*Z*,8*Z*,11*Z*,14*Z*-eicosatetraenoic acid-d8 |
| Docosahexaenoic Acid-d5 (C22:6-w3-d5) | 4*Z*,7*Z*,10*Z*,13*Z*,16*Z*,19*Z*-docosahexaenoic acid-d5 |
| Linoleic Acid-d4 (C18:2-w6-d4) | 9Z,12Z-octadecadienoic acid-d4 |
| d11-5-iPF2a-VI | (8β)-5,9α,11α-trihydroxy-prosta-6*E*,14*Z*-dien-1-oic acid-d11 |
| d4-8-iso-PGE2 | 9-oxo-11α,15*S*-dihydroxy-(8β)-prosta-5*Z*,13*E*-dien-1-oic acid-d4 |
| d4-8-iso-PGF2 α | 9α,11α,15*S*-trihydroxy-(8β)-prosta-5*Z*,13*E*-dien-1-oic acid-d4 |
| d4-PGD2 | 9α,15*S*-dihydroxy-11-oxo-prosta-5*Z*,13*E*-dien-1-oic acid-d4 |
| d4-PGF2 α | 9*S*,11*R*,15*S*-trihydroxy-5*Z*,13*E*-prostadienoic acid-d4 |
| d9-PGE2 | 9-oxo-11*R*,15*S*-dihydroxy-5*Z*,13*E*-prostadienoic acid-d9 |
| d4-iPF2 α -IV | (8*S*)-10-[(1*R*,2*S*,3*S*,5*R*)-3,5-Dihydroxy-2-pentylcyclopentyl]-8-hydroxydeca-5,9-dienoic acid-d4 |
| d11-8,12-iso-iPF2 α -VI | (12α)-5,9α,11α-trihydroxy-prosta-6*E*,14*Z*-dien-1-oic acid-d11 |
| d17-10-Nitrooleate | 10-nitro,9Z,12*Z*-octadecadienoic acid-d17 |
| d11-14,15-DiHETrE | 14,15-dihydroxy-5*Z*,8*Z*,11*Z*-eicosatrienoic acid-d11 |
| d4-9(S)-HODE | 9*S*-hydroxy-10*E*,12*Z*-octadecadienoic acid-d4 |
| d4-LTB4 | 5*S*,12*R*-dihydroxy-6*Z*,8*E*,10*E*,14*Z*-eicosatetraene-1,20-dioic acid-d4 |
| d4-TXB2 | 9*S*,11,15*S*-trihydroxy-thromboxa-5*Z*,13*E*-dien-1-oic acid-d4 |
| d6-20-HETE | 20-hydroxy-5*Z*,8*Z*,11*Z*,14*Z*-eicosatetraenoic acid-d6 |
| d8-12(S)-HETE | 12*S*-hydroxy-5*Z*,8*Z*,10*E*,14*Z*-eicosatetraenoic acid-d8 |
| d8-5(S)-HETE | 5*S*-hydroxy-6*E*,8*Z*,11*Z*,14*Z*-eicosatetraenoic acid-d8 |
| d4-(+/-)12,13-DiHOME | 12,13-dihydroxy-9*Z*-octadecenoic acid -d4 |
| d8-2-AG | 2-(5Z,8Z,11Z,14Z-eicosatetraenoyl)-sn-glycerol-d8 |
| d8-AEA | N-(5Z,8Z,11Z,14Z-eicosatetraenoyl)-ethanolamine-d8 |
| d4-COR | 11β,17,21-trihydroxypregn-4-ene-3,20-dione-d4 |
| d4-DHEA | N-(4Z,7Z,10Z,13Z,16Z,19Z-docosahexaenoyl)-ethanolamine-d4 |
| d4-LEA | N-(9Z,12Z-octadecadienoyl)-ethanolamine-d4 |
| d4-OEA | N-(9Z-octadecenoyl)-ethanolamine-d4 |
| d4-PEA | N-hexadecanoyl-ethanolamine-d4 |
| d3-SEA | N-(Octadecanoyl)-ethanolamine-d3 |

| **Table S4.** Baseline levels of oxylipins, endocannabinoids, and endocannabinoids analogues measured. | | | | | | | | | | | |
| --- | --- | --- | --- | --- | --- | --- | --- | --- | --- | --- | --- |
|  |  | **Acute effect of exercise** | | | | **Long-term effect of exercise** | | | | | |
|  |  | **Endurance**  **(n=14)** | | **Resistance**  **(n=17)** | | **CON**  **(n=36)** | | **MOD-EX**  **(n=33)** | | **VIG-EX**  **(n=33)** | |
| Group | Oxylipin | Mean | SD | Mean | SD | Mean | SD | Mean | SD | Mean | SD |
| Omega-6 oxylipins | | | | | | | | | | | |
| *LA-derived oxylipins* | LA | -3.75 | 1.01 | -3.46 | 0.76 | 1.01 | 0.07 | 1.00 | 0.09 | 1.00 | 0.11 |
|  | 9-HODE | 0.70 | 0.65 | 0.69 | 0.68 | 0.67 | 0.20 | 0.64 | 0.20 | 0.61 | 0.16 |
|  | 13-HODE | 0.46 | 0.62 | 0.24 | 0.61 | -0.02 | 0.19 | -0.05 | 0.19 | -0.10 | 0.16 |
|  | 9,10,13-TriHOME | 1.24 | 0.60 | 0.81 | 0.64 | 0.26 | 0.59 | 0.26 | 0.51 | 0.10 | 0.57 |
|  | 9,12,13-TriHOME | 0.97 | 1.63 | 0.18 | 1.68 | -0.13 | 0.51 | -0.13 | 0.56 | -0.26 | 0.48 |
|  | 9,10-EpOME | -4.62 | 1.25 | -4.75 | 1.30 | -1.31 | 0.24 | -1.40 | 0.15 | -1.44 | 0.20 |
|  | 9,10-DiHOME | - | - | - | - | 0.43 | 0.27 | 0.26 | 0.27 | 0.24 | 0.27 |
|  | 12,13-EpOME | -2.59 | 1.11 | -3.09 | 0.89 | -0.75 | 0.22 | -0.85 | 0.20 | -0.89 | 0.26 |
|  | 12,13-DiHOME | 0.38 | 0.77 | 0.13 | 0.86 | -0.07 | 0.18 | -0.12 | 0.19 | -0.14 | 0.18 |
|  | 10-NO2-LA | -4.38 | 0.60 | -4.53 | 1.18 | -1.22 | 0.26 | -1.35 | 0.31 | -1.32 | 0.30 |
|  | GLA | 3.13 | 1.25 | 3.70 | 1.16 | - | - | - | - | - | - |
| *DGLA-derived oxylipins* | DGLA | -3.83 | 0.59 | -3.33 | 0.92 | -0.06 | 0.23 | -0.08 | 0.23 | -0.07 | 0.22 |
|  | 5-HETrE | -5.21 | 0.29 | -5.21 | 0.54 | - | - | - | - | - | - |
|  | 8-HETrE | -2.93 | 0.44 | -2.92 | 0.81 | -1.15 | 0.26 | -1.10 | 0.29 | -1.09 | 0.24 |
|  | 15-HETrE | -5.89 | 0.57 | -5.68 | 0.81 | -1.82 | 0.18 | -1.87 | 0.17 | -1.85 | 0.15 |
| *AA-derived oxylipins* | AA | 1.36 | 0.45 | 1.72 | 0.51 | 1.78 | 0.12 | 1.78 | 0.15 | 1.82 | 0.16 |
|  | PGE2 | -4.15 | 1.14 | -4.14 | 1.38 | -1.24 | 0.32 | -1.30 | 0.35 | -1.20 | 0.31 |
|  | PGF2α | -5.75 | 0.08 | -6.01 | 1.15 | - | - | - | - | - | - |
|  | 2,3-dinor-8-iso-PGF2α | -7.74 | 0.72 | -7.72 | 0.83 | - | - | - | - | - | - |
|  | 13,14-dihydro-15-keto-PGF2α | -9.53 | 0.66 | -9.28 | 0.92 | - | - | - | - | - | - |
|  | TxB2 | -1.18 | 2.23 | -1.03 | 1.62 | -0.56 | 0.42 | -0.69 | 0.57 | -0.54 | 0.42 |
|  | 12-HHTrE | -2.28 | 1.30 | -2.70 | 1.61 | -0.86 | 0.35 | -0.96 | 0.48 | -0.83 | 0.39 |
|  | 8,12-IPF2α-IV | -5.91 | 0.34 | -5.97 | 0.33 | -1.66 | 0.10 | -1.66 | 0.11 | -1.66 | 0.11 |
|  | 5-HETE | -4.57 | 0.59 | -4.09 | 0.76 | -1.25 | 0.21 | -1.19 | 0.21 | -1.19 | 0.20 |
|  | 8-HETE | -4.83 | 0.28 | -4.63 | 0.51 | - | - | - | - | - | - |
|  | 9-HETE | -1.51 | 0.79 | -1.56 | 1.33 | - | - | - | - | - | - |
|  | 11-HETE | -1.49 | 0.82 | -1.58 | 1.33 | -0.67 | 0.15 | -0.67 | 0.16 | -0.64 | 0.15 |
|  | 12-HETE | -1.52 | 0.98 | -1.49 | 1.07 | -0.70 | 0.27 | -0.72 | 0.25 | -0.68 | 0.31 |
|  | 15-HETE | -4.18 | 0.48 | -3.98 | 0.60 | -1.19 | 0.13 | -1.20 | 0.13 | -1.19 | 0.14 |
|  | 20-HETE | -4.22 | 0.54 | -4.09 | 0.65 | -0.97 | 0.13 | -0.97 | 0.18 | -0.99 | 0.17 |
|  | 5,6-DiHETrE | -8.15 | 0.46 | -7.80 | 0.41 | -2.30 | 0.15 | -2.25 | 0.21 | -2.29 | 0.18 |
|  | 8,9-DiHETrE | -8.90 | 0.34 | -8.69 | 0.38 | -2.40 | 0.13 | -2.38 | 0.17 | -2.37 | 0.13 |
|  | 11,12-EpETrE | -11.80 | 0.48 | -11.45 | 0.62 | - | - | - | - | - | - |
|  | 11,12-DiHETrE | -6.22 | 0.24 | -6.05 | 0.51 | -1.54 | 0.08 | -1.55 | 0.14 | -1.55 | 0.11 |
|  | 14,15-EpETrE | -11.33 | 0.67 | -10.93 | 0.71 | -2.99 | 0.14 | -3.01 | 0.19 | -3.00 | 0.20 |
|  | 14,15-DiHETrE | -5.12 | 0.26 | -4.97 | 0.41 | -1.34 | 0.07 | -1.35 | 0.12 | -1.35 | 0.11 |
| *AdrA-deriv. oxylipins* | AdrA | -3.24 | 1.02 | -3.02 | 1.09 | -0.01 | 0.24 | -0.03 | 0.28 | -0.03 | 0.21 |
|  | 1a,1b-dihomo-PGF2α | -8.12 | 0.45 | -8.40 | 0.81 | -2.09 | 0.24 | -2.20 | 0.22 | -2.18 | 0.29 |
| Omega-3 oxylipins | | | | | | | | | | | |
| *LA-derived oxylipins* | ALA | 3.72 | 1.23 | 4.24 | 0.86 | 1.01 | 0.20 | 1.03 | 0.19 | 1.04 | 0.19 |
|  | 9-HOTrE | -4.68 | 0.78 | -4.52 | 1.02 | -1.49 | 0.32 | -1.51 | 0.21 | -1.53 | 0.21 |
|  | 12,13-DiHODE | -1.61 | 0.62 | -1.65 | 1.00 | -0.52 | 0.28 | -0.61 | 0.16 | -0.57 | 0.23 |
| *EPA-derived oxylipins* | EPA | -0.97 | 0.63 | -0.63 | 1.03 | 1.21 | 0.23 | 1.26 | 0.24 | 1.30 | 0.23 |
|  | 5-HEPE | -7.77 | 0.82 | -7.46 | 1.18 | -2.09 | 0.26 | -2.01 | 0.24 | -2.00 | 0.25 |
|  | 12-HEPE | -6.11 | 1.22 | -6.12 | 1.64 | -1.57 | 0.39 | -1.64 | 0.39 | -1.57 | 0.46 |
|  | 15-HEPE | -8.45 | 0.87 | -8.08 | 0.87 | - | - | - | - | - | - |
|  | 14,15-DiHETE | -9.65 | 0.47 | -9.72 | 0.63 | -2.76 | 0.19 | -2.74 | 0.20 | -2.69 | 0.17 |
|  | 17,18-DiHETE | -7.31 | 0.42 | -7.55 | 0.77 | -2.02 | 0.20 | -2.01 | 0.22 | -1.97 | 0.17 |
|  | DPA | -9.00 | 0.85 | -8.64 | 0.90 | -1.08 | 0.19 | -1.04 | 0.19 | -0.95 | 0.19 |
| *DHA-derived oxylipins* | DHA | 1.63 | 0.62 | 1.75 | 0.86 | 2.09 | 0.16 | 2.12 | 0.16 | 2.19 | 0.16 |
|  | 4-HDoHE | -5.73 | 1.18 | -5.92 | 1.43 | -1.82 | 0.22 | -1.78 | 0.29 | -1.77 | 0.24 |
|  | 7-HDoHE | -7.16 | 0.52 | -6.83 | 1.46 | - | - | - | - | - | - |
|  | 8-HDoHE | - | - | - | - | -1.78 | 0.21 | -1.73 | 0.19 | -1.68 | 0.23 |
|  | 10-HDoHE | -6.03 | 0.60 | -5.61 | 1.18 | - | - | - | - | - | - |
|  | 11-HDoHE | -5.37 | 1.24 | -5.17 | 1.59 | -1.23 | 0.37 | -1.25 | 0.36 | -1.17 | 0.40 |
|  | 13-HDoHE | -5.69 | 0.89 | -5.51 | 1.57 | -1.76 | 0.24 | -1.81 | 0.22 | -1.70 | 0.24 |
|  | 14-HDoHE | -5.01 | 1.18 | -4.78 | 1.29 | -1.40 | 0.40 | -1.45 | 0.46 | -1.40 | 0.43 |
|  | 16-HDoHE | -4.20 | 0.59 | -4.04 | 1.05 | -1.57 | 0.21 | -1.57 | 0.15 | -1.51 | 0.19 |
|  | 17-HDoHE | -4.40 | 0.48 | -4.35 | 1.04 | -1.45 | 0.20 | -1.46 | 0.16 | -1.42 | 0.21 |
|  | 20-HDoHE | -4.47 | 0.64 | -4.10 | 1.49 | -1.43 | 0.35 | -1.43 | 0.36 | -1.34 | 0.42 |
|  | 19,20-EpDPE | -9.91 | 1.11 | -9.88 | 1.05 | -2.68 | 0.22 | -2.64 | 0.18 | -2.60 | 0.18 |
|  | 19,20-DiHDPA | -5.30 | 0.53 | -5.47 | 0.76 | -1.53 | 0.17 | -1.51 | 0.17 | -1.49 | 0.15 |
| *Endocannabinoids and their analogues* | | | | | | | | | | | |
|  | AEA | -3.17 | 0.38 | -2.85 | 0.46 | -0.91 | 0.13 | -0.88 | 0.14 | -0.91 | 0.16 |
|  | 2-AG | 0.23 | 0.53 | 0.27 | 0.76 | -2.18 | 0.61 | -2.17 | 0.48 | -2.16 | 0.43 |
|  | 2-LG | - | - | - | - | -1.90 | 0.88 | -2.08 | 0.77 | -2.06 | 0.54 |
|  | 2-OG | - | - | - | - | -3.17 | 0.89 | -3.21 | 0.80 | -3.34 | 0.59 |
|  | DHEA | -5.02 | 0.53 | -4.78 | 0.61 | -1.16 | 0.30 | -1.20 | 0.13 | -1.18 | 0.12 |
|  | DGLEA | -6.81 | 0.62 | -6.54 | 0.65 | -1.68 | 0.20 | -1.65 | 0.16 | -1.70 | 0.18 |
|  | LEA | -2.67 | 0.41 | -2.32 | 0.41 | -0.69 | 0.15 | -0.70 | 0.15 | -0.75 | 0.14 |
|  | α-LEA | -7.36 | 0.36 | -7.09 | 0.37 | -2.18 | 0.15 | -2.20 | 0.14 | -2.18 | 0.16 |
|  | PEA | -0.91 | 0.29 | -0.55 | 0.36 | 0.23 | 0.06 | 0.23 | 0.06 | 0.22 | 0.05 |
|  | PDEA | -6.19 | 0.39 | -6.16 | 0.65 | -1.65 | 0.12 | -1.65 | 0.15 | -1.64 | 0.13 |
|  | POEA | -2.82 | 0.72 | -2.37 | 1.07 | -0.72 | 0.33 | -0.66 | 0.29 | -0.68 | 0.19 |
|  | OEA | -1.11 | 0.30 | -0.74 | 0.42 | -0.18 | 0.11 | -0.18 | 0.13 | -0.20 | 0.11 |
|  | SEA | -0.20 | 0.28 | 0.14 | 0.42 | 0.10 | 0.07 | 0.08 | 0.07 | 0.10 | 0.07 |

Data are represented as mean and standard deviation (SD) of the log2 area peak ratio of each parameter. *Abbreviations*: CON, control group: MOD-EX, moderate-intensity exercise group; VIG-EX, vigorous-intensity exercise group

| **Table S5.** Changes in oxylipins, endocannabinoids and their analogues after an endurance exercise session. | | | | | | | | | | | | | | | | |
| --- | --- | --- | --- | --- | --- | --- | --- | --- | --- | --- | --- | --- | --- | --- | --- | --- |
|  | **Baseline** | | | **3min** | | | **30min** | | | **60min** | | | | **120min** | | |
|  | Mean | SE | 95% CI | Mean | SE | 95% CI | Mean | SE | 95% CI | Mean | SE | 95% CI | Mean | | SE | 95% CI |
| *Oxylipins* |  |  |  |  |  |  |  |  |  |  |  |  |  | |  |  |
| LA-derived oxylipins | -1.586 | 1.536 | [-14.906; -8.267] | -11.630 | 1.208 | [-14.241; -9.019] | -14.875 | 1.407 | [-17.914; -11.836] | -15.004 | 1.387 | [-18.000; -12.007] | -16.365 | | 1.165 | [-18.883; -13.847] |
| DGLA-derived oxylipins | -17.858 | 0.337 | [-18.585; -17.130] | -16.917 | 0.365 | [-17.706; -16.128] | -17.554 | 0.383 | [-18.380; -16.728] | -16.600 | 0.496 | [-17.670; -15.529] | -16.285 | | 0.510 | [-17.388; -15.183] |
| AA-derived oxylipins | -107.726 | 0.1966 | [-112.010; -103.442] | -101.450 | -2.563 | [-107.034; -95.866] | -102.989 | 1.558 | `-106.384; -99.594] | -99.686 | 3.067 | [-106.367; -93.004] | -98.895 | | 2.671 | [-104.714; -93.076] |
| AdrA-derived oxylipins | -11.365 | 0.325 | [-12.068; -10.663] | -11.527 | 0.347 | [-12.276; -10.777] | -11.152 | 0.288 | [-11.774; -10.531] | -11.351 | 0.327 | [-12.057; -10.645] | -10.741 | | 0.264 | [-11.311; -10.172] |
| ALA-derived oxylipins | -2.568 | 0.477 | [-3.597; -1.538] | -1.443 | 0.501 | [-2.526; -0.360] | -2.710 | 0.665 | [-4.146; 1.274] | -2.890 | 0.674 | [-4.347; -1.433] | -2.560 | | 0.612 | [-3.881; -1.238] |
| EPA-derived oxylipins | -40.254 | 0.686 | [-41.736; -38.773] | -37.563 | 0.835 | [-39.367; -35.759] | -38.889 | 0.742 | [-40.491; -37.287] | -38.453 | 1.009 | [-40.632; -36.273] | -37.290 | | 1.049 | [-39.556; -35.024] |
| DHA-derived oxylipins | -110.763 | 1.998 | [-115.214; -106.312] | -105.650 | 3.884 | [114.303; -96.997] | -110.429 | 3.131 | [-117.406; -103.452] | -104.372 | 4.467 | [-114.324; -94.420] | -104.021 | | 4.620 | [-114.314; -93.728] |
| Ratio omega-6/3-derived oxylipins | 1.002 | 0.018 | [0.963; 1.041] | 1.016 | 0.20 | [0.971; 1.061] | 1.025 | 0.018 | [0.984; 1.006] | 1.038 | 0.017 | [1.001; 1.075] | 1.058 | | 0.028 | [0.995; 1.121] |
| *Endocannabinoids and their analogues* | |  |  |  |  |  |  |  |  |  |  |  |  | |  |  |
| AEA | -3.167 | 0.102 | [-3.388; -2.946] | -2.962 | 0.114 | [-3.208; -2.715] | -3.122 | 0.109 | [-3.357; -2.887] | -2.920 | 0.072 | [-3.075; -2.764] | -2.815 | | 0.100 | [-3.030; -2.600] |
| 2-AG | 0.228 | 0.143 | [-0.081; 0.537] | 0.567 | 0.191 | [0.155; 0.978] | 0.247 | 0.132 | [-0.038; 0.532] | 0.117 | 0.125 | [-0.153; 0.386] | -0.048 | | 0.157 | [-0.388; 0.292] |
| DHEA | -5.022 | 0.141 | [-5.327; -4.718] | -4.810 | 0.160 | [-5.155; -4.465] | -4.844 | 0.122 | [-5.107; -4.581] | -4.710 | 0.119 | [-4.967; -4.454] | -4.633 | | 0.132 | [-4.918; -4.348] |
| DGLEA | -6.811 | 0.165 | [-7.167; -6.455] | -6.584 | 0.135 | [-6.876; -6.293] | -6.595 | 0.146 | [-6.910; -6.280] | -6.501 | 0.147 | [-6.818; -6.184] | -6.464 | | 0.144 | [-6.776; -6.152] |
| LEA | -2.670 | 0.109 | [-2.906; -2.435] | -2.430 | 0.080 | [-2.602; -2.259] | -2.506 | 0.124 | [-2.773; -2.239] | -2.379 | 0.108 | [-2.612; -2.147] | -2.381 | | 0.103 | [-2.604; -2.158] |
| α-LEA | -7.356 | 0.097 | [-7.565; -7.146] | -7.161 | 0.092 | [-7.360; -6.962] | -7.252 | 0.078 | [-7.422; -7.083] | -7.223 | 0.082 | [-7.401; -7.046] | -7.177 | | 0.083 | [-7.356; -6.999] |
| PEA | -0.905 | 0.077 | [-1.071; -0.739] | -0.736 | 0.065 | [-0.875; -0.596] | -0.645 | 0.088 | [-0.836; -0.455] | -0.549 | 0.075 | [-0.710; -0.388] | -0.450 | | 0.065 | [-0.591; -0.309] |
| PDEA | -6.193 | 0.105 | [-6.420; -5.967] | -5.804 | 0.148 | [-6.124; -5.483] | -6.080 | 0.150 | [-6.403; 5.757] | -5.914 | 0.112 | [-6.156; -5.671] | -6.064 | | 0.135 | [-6.356; -5.773] |
| POEA | -2.816 | 0.193 | [-3.233; -2.400] | -2.112 | 0.155 | [-2.447; -1.778] | -2.257 | 0.202 | [-2.693; -1.820] | -2.046 | 0.197 | [-2.470; -1.621] | -1.928 | | 0.219 | [-2.400; -1.455] |
| OEA | -1.108 | 0.081 | [-1.283; -0.933] | -0.778 | 0.078 | [-0.947; -0.610] | -0.876 | 0.082 | [-1.054; -0.698] | -0.841 | 0.085 | [-1.025; -0.657] | -0.695 | | 0.073 | [-0.854; -0.537] |
| SEA | -0.203 | 0.075 | [-0.366; -0.040] | -0.043 | 0.086 | [-0.229; 0.143] | 0.153 | 0.071 | [0.000; 0.305] | 0.187 | 0.100 | [-0.030; 0.404] | 0.272 | | 0.080 | [0.100; 0.443] |

Data is presented as mean estimates, standard error (SE) and 95% confidence interval (CI) in each time point. Values obtained from repeated measures analyses of variance (ANOVA). *Abbreviations:* 2-AG, 2-arachidonylglycerol; AA, arachidonic acid; AdrA, adrenic acid; AEA, anandamide; ALA, α-linolenic acid; CON, control group; DGLA, dihomo-γ-linolenic acid; DGLEA, dihomo-gamma-linolenoyl ethanolamide; DHA, Docosahexaenoic acid; DHEA, docosahexaenoyl ethanolamide; ECBs, endocannabinoids; EPA, eicosapentaenoic acid; LA, linoleic acid; LEA, linoleoyl ethanolamide; MOD-EX, moderate intensity exercise group; OEA, oleoyl ethanolamine; PDEA, pentadecanoyl ethanolamide; PEA, palmitoyl ethanolamide; POEA, palmitoleoyl ethanolamide; SEA, stearoyl Ethanolamide; VIG-EX, vigorous intensity exercise group; α-LEA, α-Linolenoyl ethanolamide.

| **Table S6.**  Changes in oxylipins, endocannabinoids and their analogues after a resistance exercise session. | | | | | | | | | |  | | |  | | |
| --- | --- | --- | --- | --- | --- | --- | --- | --- | --- | --- | --- | --- | --- | --- | --- |
|  | **Baseline** | | | **3min** | | | **30min** | | | **60min** | | | **120min** | | |
|  | Mean  difference | SE | 95% CI | Mean  difference | SE | 95% CI | Mean  difference | SE | 95% CI | Mean  difference | SE | 95% CI | Mean  difference | SE | 95% CI |
| *Oxylipins* |  |  |  |  |  |  |  |  |  |  |  |  |  |  |  |
| LA-derived oxylipins | -13.754 | 1.458 | [-16.846; -10.663] | -13.746 | 1.290 | [-16.480; -11.012] | -15.455 | 1.248 | [-18.100; -12.810] | -14.089 | 1.225 | [-16.685; -11.492] | -13.222 | 1.340 | [-16.062; -10.381] |
| DGLA-derived oxylipins | -17.135 | 0.574 | [-18.351; -15.919] | -16.918 | 0.506 | [-17.991; -15.845] | -16.698 | 0.503 | [-17.765; -15.631] | -16.095 | 0.435 | [-17.018; -15.172] | -15.254 | 0.574 | [-16.471; 14.036] |
| AA-derived oxylipins | -106.423 | 2.916 | [-112.605; -100.241] | -102.210 | 2.419 | [-107.337; -97.083] | -100.754 | 2.019 | [-105.033; -96.474] | -97.255 | 2.018 | [-101.534; -92.977] | -94.479 | 2.443 | [-99.657; -89.301] |
| AdrA-derived oxylipins | -11.414 | 0.344 | [-12.144; -10.684] | -11.283 | 0.287 | [-11.891; -10.676] | -11.148 | 0.281 | [-11.744; -10.553] | -11.287 | 0.275 | [-11.871; -10.703] | -10.856 | 0.275 | [-11.438; -10.274] |
| ALA-derived oxylipins | -1.937 | 0.479 | [-2.953; -0.921] | -1.753 | 0.499 | [-2.811; -0.695] | -2.172 | 0.589 | [-3.420; -0.924] | -1.937 | 0.583 | [-3.172; -0.701] | -1.562 | 0.569 | [-2.769; -0.356] |
| EPA-derived oxylipins | -39.563 | 1.208 | [-42.125; -37.001] | -38.767 | 1.050 | [-40.994; -36.540] | -39.070 | 1.142 | [-41.492; -36.649] | -37.726 | 0.773 | [-39.365; -36.087] | -36.301 | 0.914 | [-38.238; -34.363] |
| DHA-derived oxylipins | -60.119 | 3.225 | [-62.037; -53.202] | -58.412 | 2.376 | [-63.508; -53.317] | -58.872 | 2.342 | [63.895; -53.848] | -55.602 | 2.395 | [-60.738; -50.466] | -51.047 | 2.804 | [-57.062; -45.033] |
| Ratio omega-6/3-derived oxylipins | 1.033 | 0.032 | [0.965; 1.101] | 1.025 | 0.028 | [0.965; 1.085] | 1.029 | 0.024 | [0.977; 1.081] | 1.026 | 0.025 | [0.973; 1.079] | 1.030 | 0.027 | [0.973; 1.087] |
| *Endocannabinoids and their analogues* | |  |  |  |  |  |  |  |  |  |  |  |  |  |  |
| AEA | -2.847 | 0.112 | [-3.083; -2.610] | -2.740 | 0.101 | [-2.954; -2.526] | -2.692 | 0.131 | [-2.969; -2.416] | -2.562 | 0.109 | [-2.792; -2.332] | -2.635 | 0.100 | [-2.848; -2.422] |
| 2-AG | 0.273 | 0.184 | [-0.118; 0.663] | 0.138 | 0.206 | [-0.299; 0.575] | 0.130 | 0.146 | [-0.180; 0.441] | 0.146 | 0.152 | [-0.175; 0.467] | 0.226 | 0.130 | [-0.051; 0.502] |
| DHEA | -4.780 | 0.148 | [-5.094; -4.466] | -4.694 | 0.126 | [-4.962; -4.426] | -4.628 | 0.127 | [-4.898; -4.359] | -4.567 | 0.100 | [-4.778; -4.356] | -4.575 | 0.102 | [-4.790; -4.360] |
| DGLEA | -6.539 | 0.157 | [-6.872; -6.206] | -6.421 | 0.106 | [-6.645; -6.197] | -6.403 | 0.155 | [-6.733; -6.074] | -6.238 | 0.101 | [-6.453; -6.023] | -6.232 | 0.107 | [-6.459; -6.004] |
| LEA | -2.323 | 0.100 | [-2.534; -2.112] | -2.250 | 0.090 | [-2.442; -2.059] | -2.211 | 0.108 | [-2.440; -1.983] | -2.105 | 0.085 | [-2.285; 1.925] | -2.181 | 0.102 | [-2.397; -1.965] |
| α-LEA | -7.094 | 0.090 | [-7.285; -6.903] | -7.018 | 0.103 | [-7.237; 6.798] | -7.006 | 0.091 | [-7.198; -6.814] | -6.968 | 0.084 | [-7.145; -6.790] | -7.053 | 0.091 | [-7.246; -6.860] |
| PEA | -0.549 | 0.087 | [-0.733; -0.365] | -0.533 | 0.092 | [-0.728; -0.339] | -0.528 | 0.104 | [-0.748; -0.308] | -0.413 | 0.097 | [-0.619; -0.206] | -0.487 | 0.103 | [-0.706; -0.268] |
| PDEA | -6.161 | 0.162 | [-6.506; -5.816] | -6.045 | 0.113 | [-6.285; -5.805] | -5.920 | 0.134 | [-6.206; -5.634] | -5.923 | 0.113 | [-6.163; -5.684] | -5.974 | 0.127 | [-6.245; -5.703] |
| POEA | -2.365 | 0.259 | [-2.914; -1.816] | -2.174 | 0.239 | [-2.680; -1.668] | -2.066 | 0.264 | [-2.625; -1.506] | -1.875 | 0.242 | [-2.387; -1.363] | -1.795 | 0.249 | [-2.322; 1.267] |
| OEA | -0.738 | 0.101 | [-0.952; -0.523] | -0.682 | 0.089 | [-0.871; -0.493] | -0.658 | 0.117 | [-0.905; -0.410] | -0.552 | 0.071 | [-0.702; -0.402] | -0.485 | 0.078 | [-0.649; -0.320] |
| SEA | 0.135 | 0.106 | [-0.091; 0.362] | 0.220 | 0.099 | [0.009; 0.432] | 0.099 | 0.106 | [-0.127; 0.324] | 0.234 | 0.108 | [0.003; 0.465] | 0.230 | 0.106 | [0.005; 0.455] |

Data is presented as mean estimates, standard error (SE) and 95% confidence interval (CI) in each time point. Values obtained from repeated measures analyses of variance (ANOVA). *Abbreviations:* 2-AG, 2-arachidonylglycerol; AA, arachidonic acid; AdrA, adrenic acid; AEA, anandamide; ALA, α-linolenic acid; CON, control group; DGLA, dihomo-γ-linolenic acid; DGLEA, dihomo-gamma-linolenoyl ethanolamide; DHA, Docosahexaenoic acid; DHEA, docosahexaenoyl ethanolamide; ECBs, endocannabinoids; EPA, eicosapentaenoic acid; LA, linoleic acid; LEA, linoleoyl ethanolamide; MOD-EX, moderate intensity exercise group; OEA, oleoyl ethanolamine; PDEA, pentadecanoyl ethanolamide; PEA, palmitoyl ethanolamide; POEA, palmitoleoyl ethanolamide; SEA, stearoyl Ethanolamide; VIG-EX, vigorous intensity exercise group; α-LEA, α-Linolenoyl ethanolamide.

| **Table S7.** Differences in plasma levels of oxylipins between groups after 24-weeks of exercise intervention. | | | | | | | | |  | | | |  |  |  |
| --- | --- | --- | --- | --- | --- | --- | --- | --- | --- | --- | --- | --- | --- | --- | --- |
|  | **MOD-EX vs. CON** | | | | **VIG-EX vs. CON** | | | | **MOD-EX vs. MOD-EX** | | | |  |  |  |
|  | Mean  difference | SE | 95% CI | P | Mean  difference | SE | 95% CI | P | Mean  difference | SE | 95% CI | **P** | **P time** | **P group** | **P time * group** |
| *Oxylipins* |  |  |  |  |  |  |  |  |  |  |  |  |  |  |  |
| LA | -0.028 | 0.016 | [-0.067; 0.011] | 0.259 | -0.037 | 0.016 | [-0.076; 0.002] | 0.071 | 0.009 | 0.017 | [0.031; 0.049] | 1.000 | 0.479 | 0.059 | 0.327 |
| 9-HODE | -0.056 | 0.030 | [-0.129; 0.017] | 0.196 | -0.073 | 0.030 | [-0.146; 0.000] | 0.050 | 0.017 | 0.031 | [-0.058; 0.092] | 1.000 | 0.069 | **0.041** | 0.684 |
| 13-HODE | -0.043 | 0.031 | [-0.117; 0.032] | 0.501 | -0.078 | 0.031 | [-0.152; -0.004] | **0.037** | 0.035 | 0.032 | [-0.041; 0.112] | 0.809 | **0.042** | **0.042** | 0.879 |
| 9,10,13-TriHOME | -0.150 | 0.086 | [-0.357; 0.056] | 0.242 | -0.123 | 0.086 | [-0.330; 0.084] | 0.460 | -0.028 | 0.088 | [-0.241; 0.186] | 1.000 | **<0.001** | 0.171 | 0.075 |
| 9,12,13-TriHOME | -0.077 | 0.078 | [-0.266; 0.112] | 0.975 | -0.074 | 0.078 | [-0.262; 0.115] | 1.000 | -0.003 | 0.081 | [-0.198; 0.192] | 1.000 | **0.002** | 0.530 | 0.270 |
| 9,10-EpOME | -0.050 | 0.036 | [-0.137; 0.037] | 0.492 | -0.103 | 0.036 | [-0.190; -0.017] | **0.013** | 0.053 | 0.037 | [-0.036; 0.142] | 0.455 | 0.458 | **0.017** | 0.399 |
| 9,10-DiHOME | -0.142 | 0.049 | [-0.260; -0.023] | **0.013** | -0.160 | 0.049 | [-0.278; -0.041] | **0.004** | 0.018 | 0.050 | [-0.104; 0.140] | 1.000 | **0.026** | **0.002** | 0.695 |
| 12,13-EpOME | -0.050 | 0.042 | [-0.152; 0.052] | 0.719 | -0.119 | 0.042 | [-0.221; -0.017] | **0.016** | 0.069 | 0.044 | [-0.036; 0.174] | 0.342 | 0.854 | **0.020** | 0.540 |
| 12,13-DiHOME | -0.056 | 0.032 | [-0.133; 0.021] | 0.240 | -0.078 | 0.032 | [-0.155; -0.002] | **0.044** | 0.022 | 0.033 | [-0.057; 0.101] | 1.000 | 0.143 | **0.040** | 0.969 |
| 10-NO2-LA | -0.086 | 0.048 | [-0.201; 0.030] | 0.223 | -0.034 | 0.048 | [-0.150; 0.081] | 1.000 | -0.052 | 0.049 | [-0.170; 0.067] | 0.890 | 0.421 | 0.201 | 0.400 |
| DGLA | -0.051 | 0.037 | [-0.141; 0.038] | 0.503 | -0.021 | 0.037 | [-0.111; 0.069] | 1.000 | -0.031 | 0.038 | [-0.123; 0.062] | 1.000 | 0.152 | 0.384 | 0.603 |
| 8-HETrE | -0.002 | 0.042 | [-0.104; 0.101] | 1.000 | 0.075 | 0.042 | [-0.027; 0.178] | 0.230 | -0.077 | 0.044 | [0.183; 0.028] | 0.238 | **0.013** | 0.129 | 0.258 |
| 15-HETrE | -0.075 | 0.029 | [-0.144; -0.005] | **0.030** | -0.050 | 0.029 | [-0.119; 0.019] | 0.251 | -0.025 | 0.030 | [-0.096; 0.047] | 1.000 | **0.031** | **0.030** | 0.518 |
| AA | -0.030 | 0.023 | [-0.086; 0.027] | 0.614 | 0.003 | 0.023 | [-0.053; 0.059] | 1.000 | -0.033 | 0.024 | [-0.091; 0.025] | 0.526 | 0.093 | 0.320 | 0.244 |
| PGE2 | -0.128 | 0.051 | [-0.252; -0.004] | **0.041** | -0.024 | 0.052 | [-0.148; 0.101] | 1.000 | -0.104 | 0.053 | [-0.233; 0.025] | 0.158 | **0.005** | **0.036** | 0.343 |
| TxB2 | -0.145 | 0.072 | [-0.319; 0.028] | 0.133 | -0.019 | 0.072 | [-0.193; 0.154] | 1.000 | -0.126 | 0.074 | [-0.305; 0.053] | 0.273 | 0.260 | 0.099 | 0.857 |
| 12-HHTrE | -0.129 | 0.062 | [-0.280; 0.021] | 0.119 | -0.005 | 0.062 | [-0.156; 0.146] | 1.000 | -0.124 | 0.064 | [-0.280; 0.031] | 0.167 | 0.125 | 0.074 | 0.801 |
| 8,12-IPF2α-IV | 0.001 | 0.016 | [-0.037; 0.040] | 1.000 | 0.021 | 0.016 | [-0.017; 0.059] | 0.557 | -0.020 | 0.016 | [-0.059; 0.020] | 0.688 | 0.538 | 0.347 | 0.352 |
| 5-HETE | -0.033 | 0.033 | [-0.114; 0.048] | 0.979 | 0.004 | 0.033 | [-0.077; 0.084] | 1.000 | -0.037 | 0.035 | [-0.120; 0.047] | 0.872 | **0.030** | 0.502 | **0.036** |
| 11-HETE | -0.051 | 0.023 | [-0.108; 0.005] | 0.086 | -0.002 | 0.023 | [-0.059; 0.054] | 1.000 | -0.049 | 0.024 | [-0.107; 0.009] | 0.130 | **<0.001** | 0.053 | 0.158 |
| 12-HETE | -0.042 | 0.046 | [-0.153; 0.068] | 1.000 | 0.011 | 0.046 | [-0.100; 0.121] | 1.000 | -0.053 | 0.047 | [-0.167; 0.061] | 0.783 | 0.288 | 0.490 | 0.864 |
| 15-HETE | -0.059 | 0.021 | [-0.110; -0.007] | **0.021** | -0.020 | 0.021 | [-0.072; 0.031] | 1.000 | -0.038 | 0.022 | [-0.092; 0.015] | 0.260 | **<0.001** | **0.024** | 0.080 |
| 20-HETE | -0.018 | 0.028 | [-0.085; 0.049] | 1.000 | -0.023 | 0.028 | [-0.090; 0.044] | 1.000 | 0.004 | 0.029 | [-0.065; 0.073] | 1.000 | 0.157 | 0.682 | 0.777 |
| 5,6-DiHETrE | 0.011 | 0.029 | [-0.059; 0.082] | 1.000 | -0.006 | 0.029 | [-0.077; 0.064] | 1..000 | 0.018 | 0.030 | [-0.055; 0.090] | 1.000 | 0.194 | 0.841 | 0.309 |
| 8,9-DiHETrE | -0.008 | 0.024 | [-0.067; 0.051] | 1.000 | 0.019 | 0.024 | [-0.040; 0.078] | 1.000 | -0.027 | 0.025 | [-0.067; 0.051] | 0.864 | 0.161 | 0.550 | 0.536 |
| 11,12-DiHETrE | -0.036 | 0.019 | [-0.083; 0.010] | 0.174 | -0.010 | 0.019 | [-0.056; 0.036] | 1.000 | -0.027 | 0.020 | [-0.074; 0.021] | 0.537 | 0.203 | 0.151 | 0.299 |
| 14,15-EpETrE | -0.018 | 0.029 | [-0.087; 0.052] | 1.000 | 0.006 | 0.028 | [-0.063; 0.074] | 1.000 | -0.023 | 0.030 | [-0.095; 0.048] | 1.000 | 0.492 | 0.715 | 0.951 |
| 14,15-DiHETrE | -0.040 | 0.018 | [-0.082; 0.003] | 0.080 | -0.047 | 0.018 | [-0.060; 0.026] | 1.000 | -0.023 | 0.018 | [-0.067; 0.022] | 0.655 | 0.112 | 0.085 | 0.187 |
| AdrA | -0.069 | 0.042 | [-0.171; 0.032] | 0.297 | -0.033 | 0.042 | [-0.134; 0.068] | 1.000 | -0.036 | 0.043 | [-0.140; 0.068] | 1.000 | 0.119 | 0.256 | 0.513 |
| 1a,1b-dihomo-PGF2α | -0.104 | 0.045 | [-0.212; 0.004] | 0.062 | -0.079 | 0.045 | [-0.187; 0.028] | 0.231 | -0.025 | 0.046 | [-0.136; 0.086] | 1.000 | 0.348 | 0.051 | 0.954 |
| Omega-3 |  |  |  |  |  |  |  |  |  |  |  |  |  |  |  |
| ALA | -0.041 | 0.032 | [-0.119; 0.037] | 0.625 | -0.015 | 0.032 | [-0.093; 0.063] | 1.000 | -0.026 | 0.033 | [-0.107; 0.055] | 1.000 | **0.035** | 0.447 | 0.201 |
| 9-HOTrE | -0.043 | 0.040 | [-0.140; 0.054] | 0.866 | -0.034 | 0.040 | [-0.131; 0.063] | 1.000 | -0.009 | 0.042 | [-0.109; 0.092 | 1.000 | 0.267 | 0.525 | 0.848 |
| 12,13-DiHODE | -0.071 | 0.036 | [-0.158; 0.015] | 0.146 | -0.054 | 0.036 | [-0.141; 0.033] | 0.406 | -0.017 | 0.037 | [-0.107; 0.072] | 1.000 | **0.002** | 0.116 | 0.798 |
| EPA | -0.023 | 0.043 | [-0.128; 0.082] | 1.000 | 0.043 | 0.043 | [-0.062; 0.147] | 0.983 | -0.066 | 0.045 | [-0.173; 0.042] | 0.431 | 0.058 | 0.331 | 0.233 |
| 5-HEPE | 0.002 | 0.046 | [-0.108; 0.112] | 1.000 | 0.020 | 0.045 | [-0.090; 0.129] | 1.000 | -0.018 | 0.047 | [-0.131; 0.095] | 1.000 | **0.032** | 0.893 | 0.184 |
| 12-HEPE | -0.056 | 0.065 | [-0.213; 0.101] | 1.000 | 0.036 | 0.065 | [-0.121; 0.193] | 1.000 | -0.092 | 0.067 | [-0.254; 0.070] | 0.517 | 0.517 | 0.387 | 0.837 |
| 14,15-DiHETE | -0.021 | 0.034 | [-0.104; 0.062] | 1.000 | 0.061 | 0.034 | [-0.022; 0.144] | 0.228 | -0.082 | 0.035 | [-0.168; 0.003] | 0.063 | 0.218 | 0.055 | 0.418 |
| 17,18-DiHETE | -0.003 | 0.035 | [-0.088; 0.082] | 1.000 | 0.060 | 0.035 | [-0.025; 0.145] | 0.267 | -0.064 | 0.036 | [-0.151; 0.024] | 0.246 | 0.804 | 0.142 | 0.828 |
| DPA | 0.007 | 0.033 | [-0.074; 0.087] | 1.000 | 0.084 | 0.033 | [0.004; 0.164] | **0.037** | -0.077 | 0.034 | [-0.160; 0.005] | 0.076 | **0.034** | **0.024** | 0.464 |
| DHA | 0.007 | 0.029 | [-0.063; 0.076] | 1.000 | 0.066 | 0.029 | [-0.004; 0.136] | 0.070 | -0.060 | 0.030 | [-0.132; 0.012] | 0.141 | 0.051 | **0.049** | 0.405 |
| 4-HDoHE | 0.001 | 0.039 | [-0.093; 0.096] | 1.000 | 0.045 | 0.039 | [-0.050; 0.140] | 0.761 | -0.044 | 0.040 | [-0.141; 0.054] | 0.844 | 0.179 | 0.443 | 0.592 |
| 8-HDoHE | -0.024 | 0.043 | [-0.128; 0.081] | 1.000 | 0.080 | 0.043 | [-0.024; 0.184] | 0.192 | -0.103 | 0.044 | [-0.210; 0.003] | 0.061 | **0.030** | 0.051 | 0.219 |
| 11-HDoHE | -0.016 | 0.062 | [-0.166; 0.133] | 1.000 | 0.044 | 0.062 | [-0.105; 0.193] | 1.000 | -0.061 | 0.064 | [-0.215; 0.093] | 1.000 | 0.640 | 0.614 | 0.917 |
| 13-HDoHE | -0.066 | 0.041 | [-0.164; 0.032] | 0.314 | 0.040 | 0.041 | [-0.058; 0.138] | 0.972 | -0.106 | 0.042 | [-0.207; -0.005] | **0.035** | 0.072 | **0.039** | 0.808 |
| 14-HDoHE | -0.054 | 0.065 | [-0.211; 0.103] | 1.000 | 0.017 | 0.065 | [-0.140; 0.174] | 1.000 | -0.071 | 0.067 | [-0.234; 0.091] | 0.868 | 0.691 | 0.539 | 0.948 |
| 16-HDoHE | -0.001 | 0.030 | [-0.073; 0.071] | 1.000 | 0.068 | 0.030 | [-0.004; 0.141] | 0.070 | -0.069 | 0.031 | [-0.144; 0.005] | 0.078 | 0.103 | **0.036** | 0.870 |
| 17-HDoHE | -0.002 | 0.032 | [-0.080; 0.076] | 1.000 | 0.074 | 0.032 | [-0.004; 0.152] | 0.067 | -0.076 | 0.033 | [-0.157; 0.004] | 0.069 | 0.340 | **0.033** | 0.338 |
| 20-HDoHE | -0.111 | 0.064 | [-0.266; 0.043] | 0.248 | -0.009 | 0.064 | [-0.162; 0.145] | 1.000 | -0.103 | 0.066 | [-0.262; 0.056] | 0.360 | 0.166 | 0.166 | 0.145 |
| 19,20-EpDPE | 0.002 | 0.035 | [-0.082; 0.086] | 1.000 | 0.061 | 0.035 | [-0.023; 0.145] | 0.237 | -0.059 | 0.035 | [-.146; 0.027] | 0.299 | 0.475 | 0.147 | 0.460 |
| 19,20-DiHDPA | -0.020 | 0.028 | [-0.087; 0.048] | 1.000 | 0.012 | 0.028 | [-0.056; 0.079] | 1.000 | -0.031 | 0.029 | [-0.101; 0.039] | 0.851 | 0.825 | 0.554 | 0.400 |

Data is presented as estimated mean difference between groups, standard error (SE) and 95% confidence interval (CI) in each time point. P time, P group and P time*group values obtained from linear mixed repeated measures analyses. P values regarding differences between groups were obtained from post-hoc Bonferroni correction..

**Table S8.** Changes in oxylipins and endocannabinoids adjusting for baseline values and changes in PUFA intake.

|  |  |
| --- | --- |
|  | **P values** |
| ΔOmega-6-derived oxylipins (log10) | **0.006** |
| ΔLA-derived oxylipins (log10) | 0.081 |
| ΔDGLA-derived oxylipins (log10) | **0.047** |
| ΔAA-derived oxylipins (log10) | **0.021** |
| ΔAdrA-derived oxylipins (log10) | 0.110 |
| ΔOmega-3-derived oxylipins (log10) | 0.285 |
| ΔALA-derived oxylipins (log10) | 0.131 |
| ΔEPA-derived oxylipins (log10) | 0.270 |
| ΔDHA-derived oxylipins (log10) | 0.241 |
| ΔRatio omega-6/3-derived oxylipins (log10) | 0.122 |
| ΔAEA (log10) | **0.002** |
| Δ2-AG (log10) | **0.008** |
| Δ2-LG (log10) | 0.185 |
| Δ2-OG (log10) | **0.011** |
| ΔDHEA (log10) | **0.018** |
| ΔDGLEA (log10) | 0.165 |
| Δα-LEA (log10) | 0.362 |
| ΔPEA (log10) | **0.024** |
| ΔPDEA (log10) | 0.074 |
| ΔPOEA (log10) | **0.001** |
| ΔOEA (log10) | **<0.001** |
| ΔSEA (log10) | 0.197 |

P value obtained from analyses of covariance (ANCOVA) adjusting for baseline values and changes in PUFA intake. *Abbreviations*: 2-AG, 2-arachidonylglycerol; AA, arachidonic acid; AdrA, adrenic acid; AEA, anandamide; ALA, α-linolenic acid; DGLA, dihomo-γ-linolenic acid; DGLEA, dihomo-gamma-linolenoyl ethanolamide; DHA, Docosahexaenoic acid; DHEA, docosahexaenoyl ethanolamide; EPA, eicosapentaenoic acid; LA, linoleic acid; LEA, linoleoyl ethanolamide; OEA, oleoyl ethanolamine; PDEA, pentadecanoyl ethanolamide; PEA, palmitoyl ethanolamide; POEA, palmitoleoyl ethanolamide; SEA, stearoyl Ethanolamide; α-LEA, α-Linolenoyl ethanolamide.

**Table S9.** Pearson correlation of 3min and 120min fold-change relative to baseline of oxylipins and endocannabinoids with basal fat oxidation and maximal fat oxidation**.**

|  | **Endurance** | | | | **Resistance** | |
| --- | --- | --- | --- | --- | --- | --- |
|  | **BFox** | | **MFO** | | **BFox** | |
|  | r | P | r | P | r | P |
| **3-min fold change relative to baseline** | | | | | | |
| *Oxylipins* | | | | | | |
| Omega-6 | 0.080 | 0.064 | 0.761 | 0.310 | 0.010 | 0.974 |
| LA | 0.220 | 0.450 | 0.185 | 0.565 | 0.250 | 0.389 |
| DGLA | 0.346 | 0.225 | -0.14 | 0.589 | 0.134 | 0.648 |
| AA | -0.104 | 0.735 | 0.285 | 0.396 | -0.069 | 0.814 |
| AdrA | -0.229 | 0.431 | 0.363 | 0.246 | -0.077 | 0.793 |
| Omega-3 | 0.370 | 0.236 | -0.179 | 0.620 | 0.402 | 0.1958 |
| ALA | 0.056 | 0.848 | -0.186 | 0.563 | 0.150 | 0.608 |
| EPA | 0.246 | 0.397 | -0.001 | 0.998 | 0.332 | 0.246 |
| DHA | 0.381 | 0.222 | -0.304 | 0.392 | 0.411 | 0.184 |
| Ratio omega 6/3 | 0.442 | 0.150 | -0.094 | 0.797 | 0.536 | 0.073 |
| *Endocannabinoids* | | | | | | |
| AEA | 0.325 | 0.256 | -0.056 | 0.863 | 0.165 | 0.574 |
| 2-AG | -0.050 | 0.865 | 0.081 | 0.801 | 0.279 | 0.334 |
| DHEA | 0.157 | 0.592 | 0.122 | 0.705 | 0.088 | 0.765 |
| DGLEA | 0.050 | 0.865 | 0.333 | 0.291 | -0.443 | 0.113 |
| LEA | 0.163 | 0.577 | -0.252 | 0.429 | 0.179 | 0.506 |
| α-LEA | -0.252 | 0.385 | 0.270 | 0.395 | 0.102 | 0.728 |
| PEA | 0.054 | 0.854 | -0.250 | 0.433 | 0.392 | 0.133 |
| PDEA | -0.113 | 0.700 | -0.100 | 0.758 | -0.078 | 0.790 |
| POEA | 0.091 | 0.758 | -0.068 | 0.834 | 0.221 | 0.411 |
| OEA | 0.377 | -0.089 | -0.106 | 0.742 | 0.038 | 0.896 |
| SEA | -0.089 | 0.762 | -0.237 | 0.459 | -0.312 | 0.240 |
| **120-min fold change relative to baseline** | | | | | | |
| *Oxylipins* | | | | | | |
| Omega-6 | 0.064 | 0.828 | 0.320 | 0.310 | 0.170 | 0.561 |
| LA | 0.165 | 0.574 | 0.254 | 0.426 | 0.463 | 0.096 |
| DGLA | 0.344 | 0.229 | 0.042 | 0.989 | -0.147 | 0.615 |
| AA | -0.018 | 0.955 | 0.607 | 0.050 | 0.255 | 0.378 |
| AdrA | -0.116 | 0.693 | 0.334 | 0.289 | -0.077 | 0.793 |
| Omega-3 | 0.298 | 0.347 | -0.246 | 0.494 | 0.402 | 0.195 |
| ALA | -0.211 | 0.469 | 0.088 | 0.787 | -0.050 | 0.866 |
| EPA | -0.070 | 0.812 | 0.252 | 0.430 | 0.332 | 0.246 |
| DHA | 0.442 | 0.150 | -0.237 | 0.510 | 0.531 | 0.076 |
| Ratio omega 6/3 | -0.080 | 0.804 | -0.144 | 0.692 | 0.161 | 0.618 |
| *Endocannabinoids* | | | | | | |
| AEA | -0.138 | 0.638 | 0.350 | 0.265 | 0.133 | 0.651 |
| 2-AG | -0.082 | 0.780 | 0.099 | 0.760 | 0.098 | 0.739 |
| DHEA | -0.006 | 0.985 | 0.142 | 0.659 | 0.087 | 0.767 |
| DGLEA | -0.039 | 0.895 | -0.089 | 0.782 | -0.166 | 0.570 |
| LEA | 0.008 | 0.979 | 0.017 | 0.959 | 0.042 | 0.886 |
| α-LEA | -0.076 | 0.797 | 0.334 | 0.288 | -0.058 | 0.843 |
| PEA | -0.309 | 0.282 | 0.189 | 0.557 | 0.668 | **0.009** |
| PDEA | 0.025 | 0.933 | 0.060 | 0.852 | 0.195 | 0.505 |
| POEA | 0.139 | 0.637 | 0.307 | 0.332 | 0.268 | 0.354 |
| OEA | -0.002 | 0.994 | 0.053 | 0.869 | 0.258 | 0.372 |
| SEA | -0.198 | 0.496 | -0.037 | 0.909 | 0.263 | 0.364 |

P value and r obtained from Pearson correlation analyses. *Abbreviations*: 2-AG, 2-arachidonylglycerol; AA, arachidonic acid; AdrA, adrenic acid; AEA, anandamide; ALA, α-linolenic acid; BFox, basal fat oxidation test; DGLA, dihomo-γ-linolenic acid; DGLEA, dihomo-gamma-linolenoyl ethanolamide; DHA, Docosahexaenoic acid; DHEA, docosahexaenoyl ethanolamide; EPA, eicosapentaenoic acid; LA, linoleic acid; LEA, linoleoyl ethanolamide; MFO, Maximal fat oxidation test, OEA, oleoyl ethanolamine; PDEA, pentadecanoyl ethanolamide; PEA, palmitoyl ethanolamide; POEA, palmitoleoyl ethanolamide; SEA, stearoyl Ethanolamide; α-LEA, α-Linolenoyl ethanolamide.

**Table S10.** Pearson correlation of changes in oxylipins and endocannabinoids with changes in basal fat oxidation and maximal fat oxidation after 24-week of exercise intervention**.**

|  | **CON (n= 36)** | | | | **MOD-EX (n= 33)** | | | | **VIG-EX (n= 33)** | | | |
| --- | --- | --- | --- | --- | --- | --- | --- | --- | --- | --- | --- | --- |
|  | **ΔBFox** | | **ΔMFO** | | **ΔBFox** | | **ΔMFO** | | **ΔBFox** | | **ΔMFO** | |
|  | r | P | r | P | r | P | r | P | r | P | r | P |
| *Oxylipins* | | | | | | |  |  |  |  |  |  |
| ΔOmega-6 | -0.021 | 0.929 | 0.275 | 0.193 | -0.172 | 0.495 | -0.141 | 0.532 | -0.104 | 0.692 | -0.306 | 0.203 |
| ΔLA | -0.071 | 0.758 | 0.321 | 0.126 | 0.048 | 0.841 | 0.352 | 0.085 | 0.223 | 0.345 | 0.016 | 0.946 |
| ΔDGLA | -0.117 | 0.604 | 0.178 | 0.394 | 0.015 | 0.950 | -0.010 | 0.963 | -0.172 | 0.455 | -0.421 | **0.045** |
| ΔAA | 0.075 | 0.741 | 0.108 | 0.609 | -0.280 | 0.261 | -0.520 | **0.009** | -0.049 | 0.837 | -0.275 | 0.215 |
| ΔAdrA | 0.119 | 0.608 | -0.188 | 0.378 | 0.082 | 0.730 | -0.210 | 0.325 | -0.542 | **0.020** | -0.440 | **0.046** |
| ΔOmega-3 | 0.059 | 0.806 | -0.019 | 0.936 | -0.303 | 0.254 | -0.158 | 0.505 | -0.124 | 0.602 | -0.126 | 0.596 |
| ΔALA | -0.179 | 0.427 | -0.074 | 0.730 | -0.111 | 0.641 | -0.058 | 0.778 | -0.119 | 0.608 | -0.193 | 0.377 |
| ΔEPA | 0.427 | **0.047** | 0.126 | 0.549 | -0.361 | 0.129 | -0.077 | 0.716 | 0.085 | 0.715 | 0.033 | 0.882 |
| ΔDHA | 0.573 | **0.008** | -0.120 | 0.605 | -0.367 | 0.147 | -0.340 | 0.131 | -0.017 | 0.945 | 0.003 | 0.990 |
| ΔRatio omega 6/3 | -0.063 | 0.805 | -0.163 | 0.506 | -0.066 | 0.822 | -0.088 | 0.738 | -0.752 | **0.001** | -0.752 | **0.001** |
| *Endocannabinoids* | | | | | | |  |  |  |  |  |  |
| ΔAEA | 0.188 | 0.403 | 0.283 | 0.170 | 0.102 | 0.668 | -0.045 | 0.826 | -0.001 | 0.996 | -0.275 | 0.203 |
| Δ2-AG | -0.303 | 0.171 | 0.231 | 0.266 | 0.204 | 0.403 | 0.185 | 0.375 | -0.373 | 0.096 | -0.081 | 0.712 |
| Δ2-LG | -0.256 | 0.251 | -0.013 | 0.951 | 0.138 | 0.563 | 0.348 | 0.082 | -0.328 | 0.147 | -0.046 | 0.836 |
| Δ2-OG | -0.211 | 0.346 | 0.057 | 0.787 | 0.187 | 0.429 | 0.189 | 0.356 | -0.352 | 0.128 | -0.071 | 0.749 |
| ΔDHEA | 0.397 | 0.075 | 0.271 | 0.200 | -0.052 | 0.827 | -0.065 | 0.754 | 0.150 | 0.516 | -0.013 | 0.955 |
| ΔDGLEA | -0.247 | 0.268 | 0.171 | 0.414 | 0.207 | 0.395 | -0.107 | 0.612 | 0.005 | 0.984 | -0.228 | 0.307 |
| ΔLEA | -0.007 | 0.976 | 0.221 | 0.288 | 0.047 | 0.843 | 0.040 | 0.848 | -0.081 | 0.728 | -0.301 | 0.162 |
| Δα-LEA | 0.163 | 0.467 | 0.165 | 0.432 | -0.271 | 0.248 | 0.001 | 0.995 | 0.030 | 0.897 | 0.018 | 0.934 |
| ΔPEA | 0.088 | 0.698 | 0.341 | 0.095 | 0.060 | 0.809 | -0.125 | 0.553 | 0.176 | 0.446 | -0.170 | 0.439 |
| ΔPDEA | 0.065 | 0.773 | 0.179 | 0.393 | -0.526 | **0.017** | 0.025 | 0.904 | 0.092 | 0.691 | 0.128 | 0.562 |
| ΔPOEA | -0.318 | 0.150 | 0.187 | 0.371 | -0.144 | 0.544 | -0.076 | 0.711 | -0.055 | 0.811 | -0.138 | 0.530 |
| ΔOEA | 0.105 | 0.642 | 0.146 | 0.487 | -0.096 | 0.687 | -0.105 | 0.608 | 0.055 | 0.812 | -0.240 | 0.270 |
| ΔSEA | -0.036 | 0.875 | 0.349 | 0.087 | -0.219 | 0.353 | -0.124 | 0.547 | -0.164 | 0.477 | -0.124 | 0.572 |

P value and r obtained from Pearson correlation analyses. *Abbreviations*: 2-AG, 2-arachidonylglycerol; AA, arachidonic acid; AdrA, adrenic acid; AEA, anandamide; ALA, α-linolenic acid; BFox, basal fat oxidation test, CON, control group; DGLA, dihomo-γ-linolenic acid; DGLEA, dihomo-gamma-linolenoyl ethanolamide; DHA, Docosahexaenoic acid; DHEA, docosahexaenoyl ethanolamide; EPA, eicosapentaenoic acid; LA, linoleic acid; LEA, linoleoyl ethanolamide; MFO, Maximal fat oxidation; MOD-EX, moderate-intensity exercise group; OEA, oleoyl ethanolamine; PDEA, pentadecanoyl ethanolamide; PEA, palmitoyl ethanolamide; POEA, palmitoleoyl ethanolamide; SEA, stearoyl Ethanolamide; VIG-EX, vigorous-intensity exercise group; α-LEA, α-Linolenoyl ethanolamide.


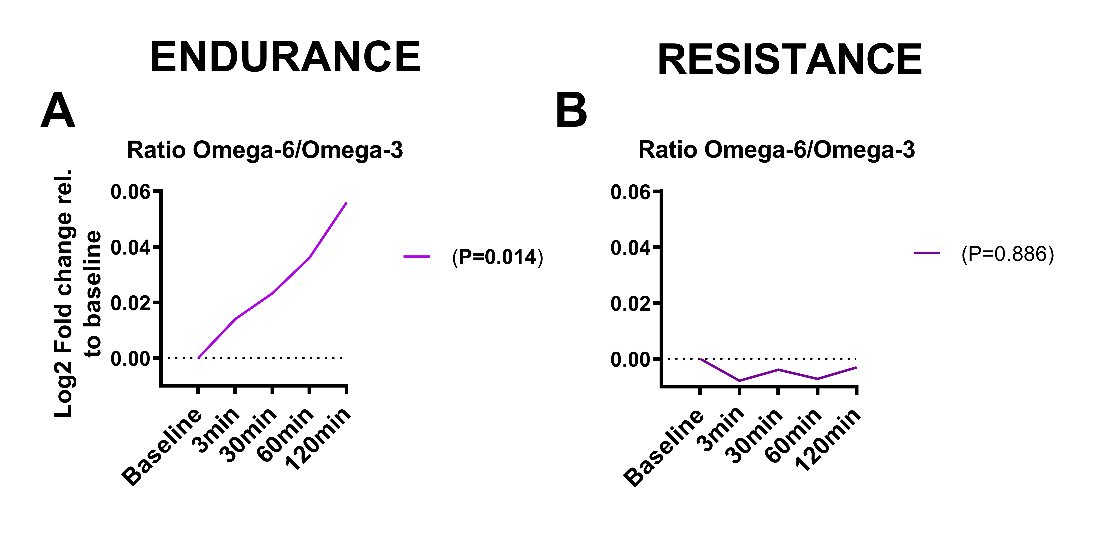


**Figure S1. Acute endurance, but not resistance, exercise increases the ratio of omega-6/omega-3 oxylipins.** Changes in the ration of omega-6/omega-3 oxylipins after acute endurance (A) and resistance (B) exercises. Each line represents the trajectory of the mean log2 fold change relative to baseline of the ratio omega-6/omega-3. P value obtained from repeated measures analyses of variance (ANOVA).


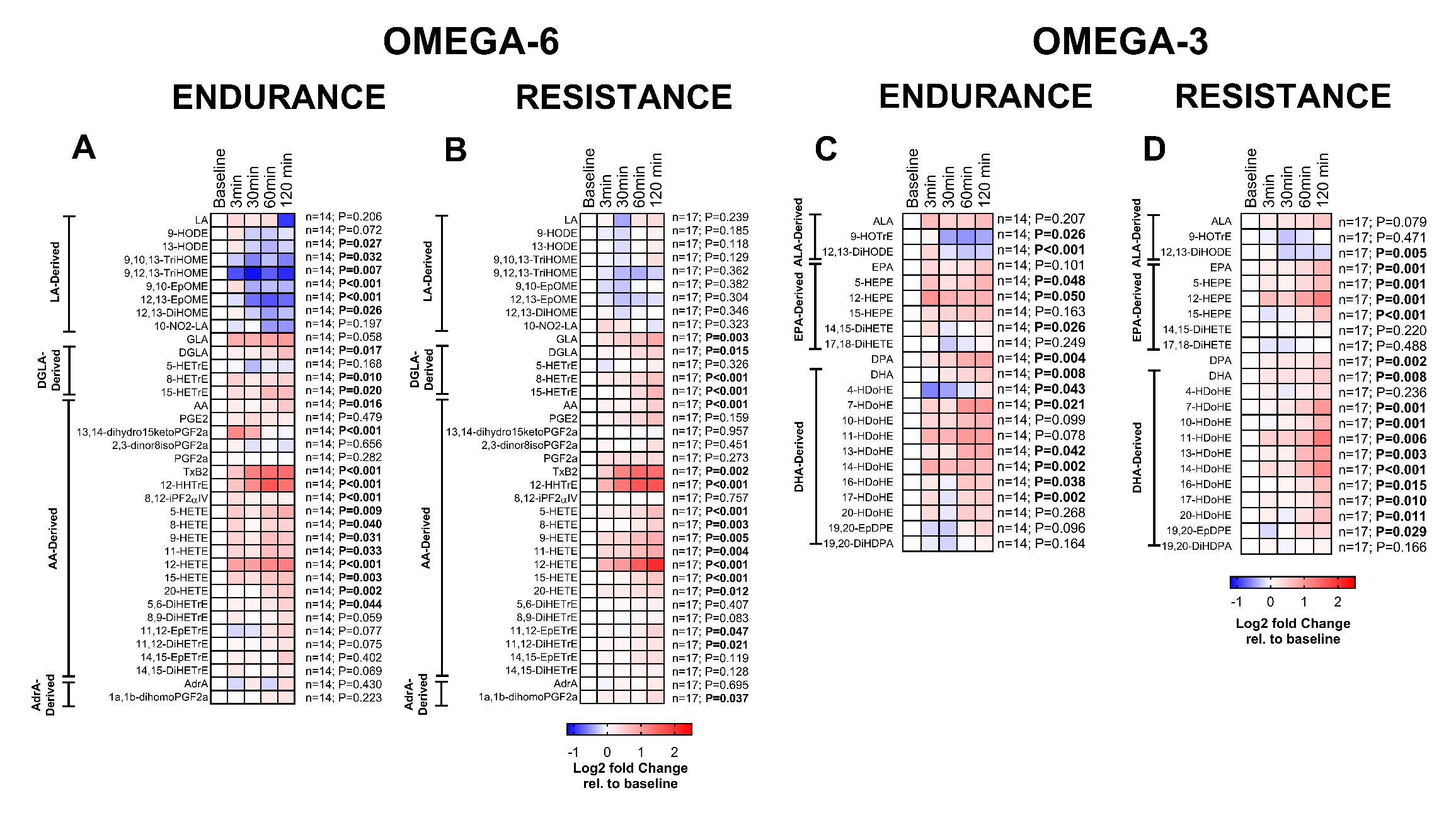


**Figure S2. Endurance and resistance exercises acutely increase plasma levels of omega-6 and omega-3 oxylipins.** The color of the squares represents the mean log2 fold change of the area peak ratio of that timepoint relative to baseline in acute endurance (A, C) and resistance (B, D) exercises. Red color represents an increase, whereas blue represents a decrease. P value obtained from repeated measures analyses of variance (ANOVA).


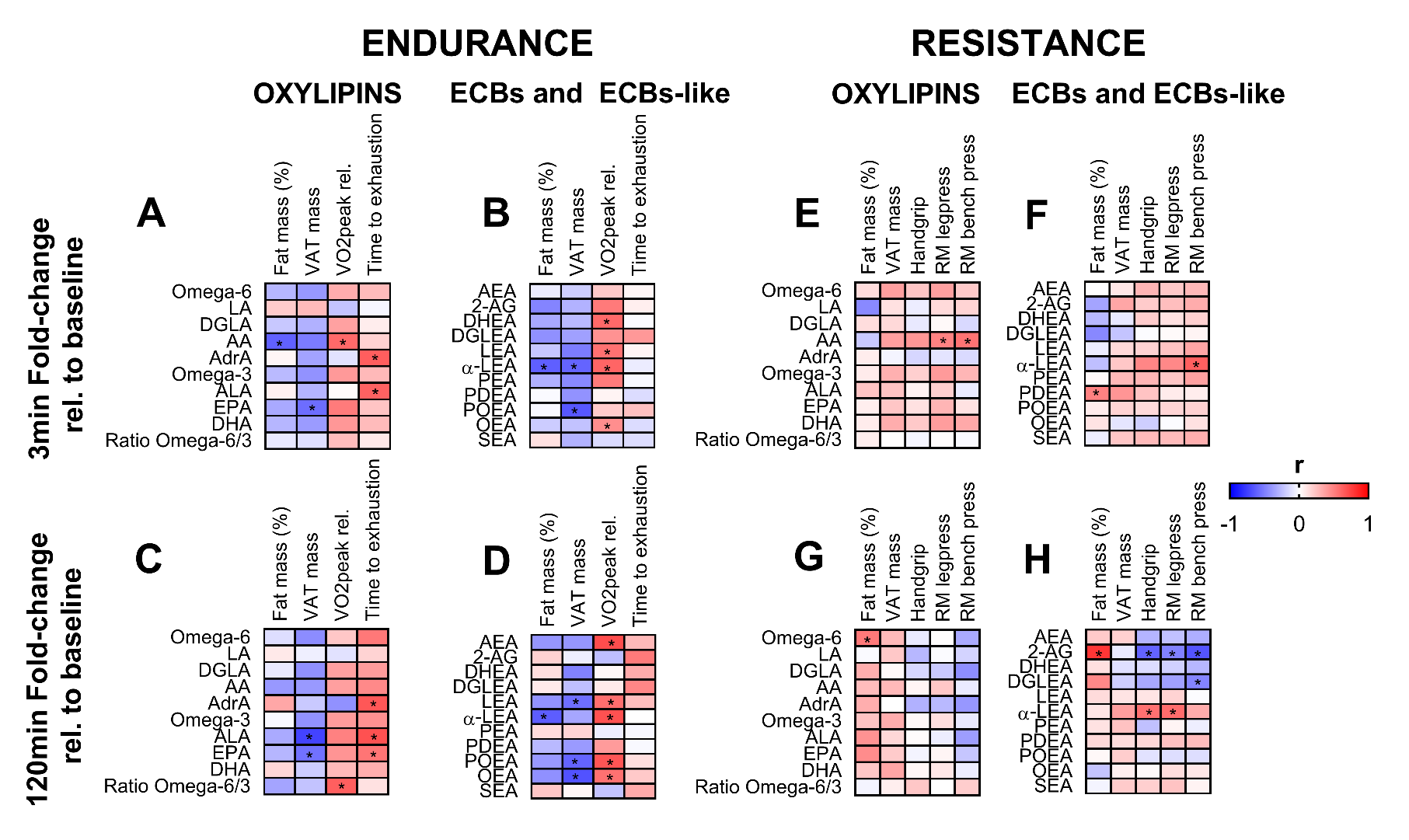


**eCBs and analogues**

**eCBs and analogues**

**Figure S3. Changes in oxylipins, endocannabinoids and their analogues after acute endurance and resistance exercises are correlated with body composition and physical fitness.** Heat map showing the Pearson correlation of 3min and 120min fold-change relative to baseline of oxylipins and endocannabinoids with adiposity and physical fitness. For the analyses the sum of LA-derived, DGLA-derived, AA-derived, AdrA-derived, ALA-derived, EPA-derived, and DHA-derived oxylipins were calculated. The color of the squares represents r of Pearson. Red color represents a positive correlation, whereas blue represents a negative correlation. * Symbol indicates significant correlations (P<0.05) between outcomes. *Abbreviations*: 2-AG, 2-arachidonylglycerol; AA, arachidonic acid; AdrA, adrenic acid; AEA, anandamide; ALA, α-linolenic acid; DGLA, dihomo-γ-linolenic acid; DGLEA, dihomo-gamma-linolenoyl ethanolamide; DHA, Docosahexaenoic acid; DHEA, docosahexaenoyl ethanolamide; ECBs, endocannabinoids; EPA, eicosapentaenoic acid; LA, linoleic acid; LEA, linoleoyl ethanolamide; OEA, oleoyl ethanolamine; PDEA, pentadecanoyl ethanolamide; PEA, palmitoyl ethanolamide; POEA, palmitoleoyl ethanolamide; RM, repetition maximum; SEA, stearoyl Ethanolamide; VAT, visceral adipose tissue; VO_2 rel._, oxygen consumption relative to body weight; VO_2_, oxygen consumption; α-LEA, α-Linolenoyl ethanolamide.
