# Supplementary material for: Acute and long-term exercise differently modulate plasma levels of oxylipins, endocannabinoids, and their analogues in young sedentary adults: A sub-study and secondary analyses from the ACTIBATE randomized controlled-trial
Source: eBioMedicine. 2022 Oct 27;85:104313. doi: 10.1016/j.ebiom.2022.104313 (PMC9626892; doi:10.1016/j.ebiom.2022.104313)
Supplement: Supplemental Tables S1–S10 and Figures S1–S3 Captions [file mmc2.docx]

***CAPTION FOR SUPPLEMENTAL MATERIAL***

**Table S1**. 2017 CONSORT checklist of information to include when reporting a randomized trial assessing nonpharmacologic treatments (NPTs).

**Table S2**. List of oxylipins, endocannabinoids, and endocannabinoids analogues measured.

**Table S3.** List of internal standards used in the LC/MS method.

**Table S4.** Baseline levels of oxylipins, endocannabinoids, and endocannabinoids analogues measured.

**Table S5.** Changes in oxylipins, endocannabinoids and their analogues after an endurance exercise sesión.

**Table S6.**  Changes in oxylipins, endocannabinoids and their analogues after a resistance exercise session.

**Table S7.** Differences in plasma levels of oxylipins between groups after 24-weeks of exercise intervention.

**Table S8.** Changes in oxylipins and endocannabinoids adjusting for baseline values and changes in PUFA intake.

**Table S9.** Pearson correlation of 3min and 120min fold-change relative to baseline of oxylipins and endocannabinoids with basal fat oxidation and maximal fat oxidation**.**

**Table S10.** Pearson correlation of changes in oxylipins and endocannabinoids with changes in basal fat oxidation and maximal fat oxidation after 24-week of exercise intervention**.**

**Figure S1.** Acute endurance, but not resistance, exercise increases the ratio of omega-6/omega-3 oxylipins.

**Figure S2.** Endurance and resistance exercises acutely increase plasma levels of omega-6 and omega-3 oxylipins.

**Figure S3.** Changes in oxylipins, endocannabinoids and their analogues after acute endurance and resistance exercises are correlated with body composition and physical fitness.
